# Macromolecular crowding in human tenocyte and skin fibroblast cultures: A comparative analysis

**DOI:** 10.1016/j.mtbio.2024.100977

**Published:** 2024-01-28

**Authors:** Adrian Djalali-Cuevas, Mandy Rettel, Frank Stein, Mikhail Savitski, Stephen Kearns, Jack Kelly, Manus Biggs, Ioannis Skoufos, Athina Tzora, Nikitas Prassinos, Nikolaos Diakakis, Dimitrios I. Zeugolis

**Affiliations:** a Laboratory of Animal Science, Nutrition and Biotechnology, School of Agriculture, University of Ioannina, Arta, Greece; b School of Veterinary Medicine, Aristotle University of Thessaloniki, Thessaloniki, Greece; c Regenerative, Modular & Developmental Engineering Laboratory (REMODEL), Charles Institute of Dermatology, Conway Institute of Biomolecular & Biomedical Research and School of Mechanical & Materials Engineering, University College Dublin (UCD), Dublin, Ireland; d Proteomics Core Facility, European Molecular Biology Laboratory (EMBL), Heidelberg, Germany; e Merlin Park University Hospital, Galway, Ireland; f Galway University Hospital, Galway, Ireland; g Science Foundation Ireland (SFI) Centre for Research in Medical Devices (CÚRAM), Biomedical Sciences Building, University of Galway, Galway, Ireland

**Keywords:** Tendon engineering, Dermal fibroblasts, Tenocytes, Macromolecular crowding, Proteomics

## Abstract

Although human tenocytes and dermal fibroblasts have shown promise in tendon engineering, no tissue engineered medicine has been developed due to the prolonged *ex vivo* time required to develop an implantable device. Considering that macromolecular crowding has the potential to substantially accelerate the development of functional tissue facsimiles, herein we compared human tenocyte and dermal fibroblast behaviour under standard and macromolecular crowding conditions to inform future studies in tendon engineering. Basic cell function analysis made apparent the innocuousness of macromolecular crowding for both cell types. Gene expression analysis of the without macromolecular crowding groups revealed expression of tendon related molecules in human dermal fibroblasts and tenocytes. Protein electrophoresis and immunocytochemistry analyses showed significantly increased and similar deposition of collagen fibres by macromolecular crowding in the two cell types. Proteomics analysis demonstrated great similarities between human tenocyte and dermal fibroblast cultures, as well as the induction of haemostatic, anti-microbial and tissue-protective proteins by macromolecular crowding in both cell populations. Collectively, these data rationalise the use of either human dermal fibroblasts or tenocytes in combination with macromolecular crowding in tendon engineering.

## Highlights

• Dermal fibroblasts express tendon regulatory genes and proteins.
• Macromolecular crowding enhances dermal fibroblast and tenocyte collagen deposition.
• Dermal fibroblasts and tenocytes produce similar in composition extracellular matrix.
• Dermal fibroblasts and tenocytes quantitative proteome show great similarity.
• Dermal fibroblasts and macromolecular crowding show promise for tendon engineering.

## Graphical abstract

Macromolecular crowding in human dermal fibroblast and human tenocyte cultures.

**Figure:**
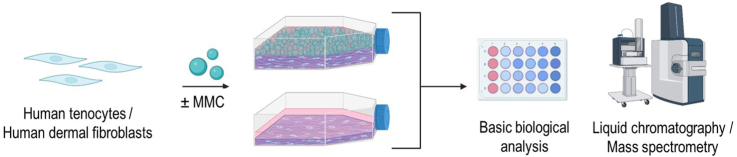

## 1 Introduction

The global prevalence of musculoskeletal disorders is in excess of 1.3 billion, which are associated with adverse patient (e.g. long-term pain, physical disability, prolonged distress, reduced quality of life) and economy (e.g. in United States US$381 billion and in Europe €240 billion healthcare costs) outcomes [1]. The annual tendon and ligament related injuries and pathophysiologies in the United States alone account for ∼2.5 % of the global musculoskeletal disorders [2], with devastating economic consequences (e.g. in United States alone, extensor tendon lacerations only account for US$300–530 million per year direct and indirect costs) [3]. Although biomaterials and tissue grafts are used extensively in clinic, preclinical data and clinical trials have revealed that they yield thinner, weaker and less functional neotissue [4]. Cell injections have also shown varied therapeutic efficiency, as the mode of administration offers little control over localisation, retention and distribution of the injected cell suspension [5]. To this end, tissue engineering strategies, using a diverse range of cell populations and microenvironmental cues [[6], [7], [8]], are emerging in tendon engineering [9,10]. Such approaches exploit the inherent capacity of cells to produce highly sophisticated supramolecular assemblies that mimic native tissue architecture and complex extracellular matrix (ECM) composition [11].

Although human tenocytes (hTCs) [[12], [13], [14], [15]] and human dermal fibroblasts (hDFs) [[16], [17], [18], [19], [20]] are used extensively in the development of tendon engineering cell-based medicines and have demonstrated even acceptable clinical results, no commercial product has been developed to-date. In the case of hDFs, the limiting factor in their commercial realisation may be their specificity [21,22]. We believe that the major roadblock in commercial translation of tissue engineered medicines is the prolonged *in vitro* culture required to develop an implantable device that is associated with very high cost of goods [[23], [24], [25]] and, in the case of hTCs, loss of native phenotype, function and therapeutic potential [26,27]. For this reason, to the best of our knowledge, scaffold-free tendon transplants have only been developed from non-human cells (e.g. rat patellar tendon stem cells, 2 weeks in culture with ascorbic acid and connective tissue growth factor [28,29]), imposing the need for the development of culture conditions that will bridge the gap between positive research outcomes and market success.

Macromolecular crowding (MMC) is a biophysical phenomenon that via volume exclusion reduces diffusion and increases the kinetics of biochemical reactions and biological processes [[30], [31], [32], [33]]. Recent studies have argued that MMC may enable the accelerated development of functional tissue engineered medicines [[34], [35], [36]]. Indeed, in both hTC [[37], [38], [39], [40]] and hDF [[41], [42], [43]] cultures, MMC has been shown to enhance (up to 120-fold) and accelerate (from 2 to 6 days) ECM deposition and to maintain hTC phenotype alone or in combination with other *in vitro* microenvironment modulators. It is also worth noting that the therapeutic potential of MMC has been demonstrated in preclinical setting [44], paving the way for its clinical translation. With the potential therefore now, with the utilisation of MMC, to develop in fast manner functional tendon facsimiles out of hTCs and hDFs, herein, an in-depth molecular profiling analysis of hTCs and hDFs was performed under standard (-MMC) and MMC (+MMC) conditions.

## 2 Materials and methods

### 2.1 Materials and reagents

Unless otherwise stated, cell culture materials were purchased from Sarstedt (Greece); chemical reagents and cell culture media and supplements were purchased from Sigma-Aldrich (Greece); and RNA isolation and reverse-transcribed real-time polymerase chain reaction (RT-qPCR) materials and reagents were purchased from Bio-Rad Laboratories (UK).

### 2.2 Cells and cell cultures

Cryopreserved hDFs (PCS-201-012) were obtained from ATCC (UK). hTCs were isolated from surgical remains of healthy tendons donated by patients (appropriate ethical approvals and informed consents were in place) undergoing tendon-related procedures at Merlin Park University Hospital (Ireland) and Galway University Hospital (Ireland), by means of an explant tissue culture approach [45]. Three different donors were used for this study. Patient sex and details for both cell types are provided in Supplementary Table S1. Routine cell culture practices and conditions were applied throughout the study. Cells were cultured in high glucose Dulbecco's modified Eagle medium, 10 % foetal bovine serum, 100 units/ml penicillin and 100 μg/ml streptomycin (basal medium) at 37 °C and 5 % CO_2_ humidified atmosphere. MMC cultures were conducted as has been described previously in detail [[46], [47], [48]] with minor modifications. Briefly, at passage 4, hTCs and hDFs were seeded in 6-, 24-, 48-, or 96- well tissue culture plates at 25,000 cells/cm^2^ and cultured in basal medium for 24 h for allowing proper cell adhesion. Subsequently, the basal medium was replaced with basal medium supplemented with 100 μM l-ascorbic acid 2-phosphate in control groups (-MMC) and with basal medium supplemented with 100 μM l-ascorbic acid 2-phosphate and 10 μg/ml of λ-carrageenan (Viscarin® GP 109 NF, FMC Corporation, USA) in MMC-treated groups (+MMC). -MMC and +MMC media were replaced every 3 days with fresh respective media and analysis was performed after 4, 7 and 10 days of the initial cell seeding. The specific MMC agent and concentration were selected on the basis of previous screening and optimisation [49].

### 2.3 Cell morphology and proliferation assessment

Cell morphology and proliferation were analysed through cytoskeletal and nuclear fluorescent imaging, respectively. At all experimental time points, cell culture media were removed and cell layers were washed three times with sterile-filtered Hank's Balanced Salt Solution (HBSS). Fixation was performed by incubation in an ice-chilled solution of 2 % paraformaldehyde (PFA) in phosphate buffered saline (PBS) for 15 min, followed by three washes with PBS. Cell layers were permeabilised for 10 min with a 0.1 % Triton X-100 solution in PBS. Blocking was performed for 1.5 h with a 5 % bovine serum albumin (BSA) and 0.02 % Triton X-100 solution in PBS. Subsequently, actin filaments (F-actin) were stained with 50 nM rhodamine-phalloidin conjugate (Invitrogen, Ireland) in a solution of 3 % BSA and 0.02 % Triton X-100 in PBS for 2.5 h. Samples were then washed three times with a solution of 0.5 % BSA and 0.02 % Triton X-100 in PBS. Samples were post-fixed with a 2 % solution of PFA in PBS for 15 min. An additional wash with 0.5 % BSA and 0.02 % Triton X-100 in PBS was carried out before staining nuclei with 0.5 μg/ml 4′,6-diamidino-2-phenylindole (DAPI) in 0.5 % BSA and 0.02 % Triton X-100 in PBS for 5 min. After three additional washes with PBS, samples were visualised with an Olympus IX73 inverted fluorescence microscope (Olympus Corporation, Japan). Cell nuclei were quantified using ImageJ software (NIH, USA).

### 2.4 Cell viability and metabolic activity assessment

Cell viability was assessed by dual fluorescent staining with a solution of 4 μM calcein-AM and 2 μM ethidium homodimer in HBSS. At all experimental time points, cell layers were washed with HBSS and incubated with staining solution for 30 min at 37 °C and 5 % CO_2_ humidified atmosphere. The staining solution was then removed and samples were washed again with HBBS before visualisation with an Olympus IX73 inverted fluorescence microscope (Olympus Corporation, Japan). Dead cells were quantified using ImageJ software (NIH, USA).

Cell metabolic activity was assessed by spectrophotometric analysis of the reduction of resazurin to resorufin. At each time point, cell layers were washed with HBSS and incubated with a 44 μM solution of resazurin in HBSS for 3 h at 37 °C and 5 % CO_2_ humidified atmosphere. HBSS and resazurin solution were also incubated in empty wells in order to obtain spectrophotometric references. After the incubation period, 100 μl from each well were transferred to the wells of a clear plate and absorbances were measured at 550 nm and 595 nm using a Biotek® Synergy™ HT microplate reader (Biotek Instruments, USA). The % of reduced resazurin was calculated from absorbance values.

### 2.5 Gene expression assessment

Analysis of the expression of tendon-, cartilage- and bone-related genes was performed via RT-qPCR. Given the potentially inhibitory effects of carrageenan (CR) in the reverse-transcription reaction [50], +MMC groups were not included in the analysis. At the end of all experimental time points, culture media were removed and total RNA was isolated with PureZOL™ RNA isolation reagent. Total RNA concentration and purity were estimated using a SmartSpec™ Plus spectrophotometer (Bio-Rad Laboratories, USA). RNA integrity was assessed using Qubit™ RNA IQ assay kit with a Qubit™ 4 fluorometer (Invitrogen, USA). RNA was reverse-transcribed using iScript™ gDNA Clear cDNA Synthesis Kit. Expression of the tendon-related genes *COL1A1*, *COL3A1*, *SCX*, *TNMD* and *MKX*, as well as the chondrogenic and osteogenic genes *SOX9* and *RUNX2*, respectively, was evaluated by real-time PCR using the SsoAdvanced™ Universal SYBR® Green Supermix in a Chromo4™ System connected to a DNA Engine® thermal cycler (Bio-Rad Laboratories, USA). Manufacturer's instructions were followed in every case. Gene expression values were calculated relative to the expression levels of each corresponding gene in hTCs at day 4 by means of the ΔCt method. Primer sequences and characteristics are detailed in Supplementary Table S2.

### 2.6 Immunofluorescent staining assessment

Cells were cultured as described above in Nunc® Lab-Tek® II Chamber Slide™ system (ThermoFisher, USA). At each time point, culture media were removed and cell layers were washed three times with HBSS. Samples were fixed with an ice-chilled solution of 2 % PFA in PBS for 15 min and washed again three times with PBS. Cell layers were permeabilised for 10 min with 0.1 % Triton X-100 solution in PBS. Blocking was performed for 1.5 h with a solution of 5 % BSA and 0.02 % Triton X-100 in PBS. Immediately after blocking, samples were incubated for 2.5 h with the corresponding primary antibodies diluted in 3 % BSA and 0.02 % Triton X-100 in PBS. When necessary, F-actin co-staining was performed through the addition of 50 nM rhodamine-phalloidin conjugate (Invitrogen, Ireland) in the corresponding primary antibody solution. After incubation with primary antibodies, samples were washed three times with 0.5 % BSA and 0.02 % Triton X-100 in PBS. After washing, samples were incubated for 30 min with the corresponding secondary antibodies diluted in 3 % BSA and 0.02 % Triton X-100 in PBS. Alexa Fluor® Plus 488 and Alexa Fluor® Plus 555 were the conjugated fluorochromes used to detect scleraxis (SCX) or tenomodulin (TNMD) and alpha-smooth muscle actin (α-SMA), respectively. Samples were washed three times with 0.5 % BSA and 0.02 % Triton X-100 in PBS before post-fixation in 2 % PFA in PBS for 15 min and washed once again with 0.5 % BSA and 0.02 % Triton X-100 in PBS. Nuclear staining was performed for 5 min with a solution of 0.5 μg/ml DAPI in 0.5 % BSA and 0.02 % Triton X-100 in PBS. After three washes with PBS, samples were visualised with an Olympus IX73 inverted fluorescence microscope (Olympus Corporation, Japan). Images were quantified using ImageJ software (NIH, USA). Detailed information of primary and secondary antibodies can be found in Supplementary Table S3. For α-SMA staining, a positive control was prepared with hDFs as per previously published work [51].

To assess the deposition of different collagen types, cell layers were washed three times with HBSS and fixed with an ice-chilled solution of 2 % PFA in PBS for 15 min at all experimental time-points. After three washes with PBS, blocking was performed with a 3 % BSA solution in PBS for 30 min. Samples were then incubated immediately with primary antibodies (Abcam, UK) diluted in PBS for 1.5 h. After three washes in PBS, samples were incubated for 30 min with the corresponding secondary antibodies. Alexa Fluor® Plus 555 was the conjugated fluorochrome used to detect collagen type I (COL I), whilst Alexa Fluor® Plus 488 was used to detect collagen types III, IV, V and VI (COL III, IV, V and VI). After three washes in PBS, post-fixation was performed for 15 min with a 2 % PFA solution in PBS. After an additional wash in PBS, nuclei were stained for 5 min with a 0.5 μg/ml DAPI solution in methanol. Samples were washed three times with PBS before being visualised with an Olympus IX73 inverted fluorescence microscope (Olympus Corporation, Japan). To compare the amounts of deposited collagen between different samples, fluorescence intensity analysis was performed using ImageJ software (NIH, USA). Detailed information of primary and secondary antibodies can be found in Supplementary Table S3.

### 2.7 Deposited collagen assessment via electrophoresis

Deposited collagen was assessed via sodium dodecyl sulphate polyacrylamide gel electrophoresis (SDS-PAGE), as has been described previously [52]. Cell layers were washed three times with HBSS and digested with a solution of 100 μg/ml pepsin from porcine gastric mucosa (>3200 U/mg, P6887, Sigma-Aldrich, Greece) and 50 mM glacial acetic acid in HBSS for 2 h at 37 °C. Cell layers were scraped from the culture surfaces immediately after incubation. Samples were neutralised with 1 M NaOH to inhibit pepsin activity. Pepsin-extracted purified bovine collagen type I (Symatese Biomateriaux, France) was used as a standard reference at 1 mg/ml in 50 mM acetic acid, neutralised as above. Samples were diluted with distilled water and SDS-PAGE sample loading buffer and heated at 95 °C for 5 min immediately before loading. Samples were run in 3 % stacking and 5 % resolving non-reducing tris-glycine SDS polyacrylamide electrophoresis gels, using the Mini-PROTEAN® Tetra Cell Electrophoresis System (Bio-Rad Laboratories, USA). Electrophoresis was carried out at 50 V for the initial 30 min and at 120 V for 1 h. The gels were stained using the PlusOne® Silver Staining Kit, Protein (GE-Healthcare, USA), according to the manufacturer's instructions. Gels were imaged using an HP ScanJet 600 desktop scanner (Hewlett-Packard, USA). The amount of collagen type I in each sample was assessed by analysing the intensities of collagen *α*1(I) and *α*2(I) chains using ImageJ (NIH, USA), with respect to the collagen type I standard sample.

### 2.8 Proteolytic activity assessment

The analysis of matrix metalloproteinases (MMPs) was performed by gelatine zymography as has been described previously [48]. In brief, 3 % stacking and 10 % resolving non-reducing tris-glycine SDS-PAGE gels containing 0.1 mg/ml type B bovine gelatine were prepared. Fresh and conditioned culture media samples were diluted with distilled water and SDS-PAGE sample loading buffer prior loading. Electrophoresis was performed at 50 V for the initial 30 min and at 120 V for 1 h, using the Mini-PROTEAN® Tetra Cell Electrophoresis System (Bio-Rad Laboratories, UK). Gels were washed with 2.5 % Triton X-100 for 1 h, rinsed briefly with distilled water and incubated for 18 h at 37 °C with a solution of 5 mM CaCl_2_, 1 μM ZnCl_2_ and 2.5 % Triton X-100 in 50 mM Tris-HCl pH 7.5. Gels were stained with a solution of 0.5 % of Coomassie Brilliant Blue G-250, 30 % methanol and 10 % acetic acid in distilled water. De-staining was performed with a solution of 30 % methanol and 10 % acetic acid in distilled water. Gels were imaged using an HP ScanJet 600 desktop scanner (Hewlett-Packard, USA). Densitometric analysis was performed using ImageJ (NIH, USA).

### 2.9 Proteomics assessment

Intact cell layers were used for protein extraction. Briefly, after 10 days of culture, cell layers were gently detached from the culture surfaces with a cell scraper and stored at −80 °C after flash-freeze in liquid N_2_. 4 % SDS in 50 mM Tris-HCl pH 7.6 was used as lysis buffer. The total protein concentration was estimated by means of the Bradford's assay using BSA as standard and the concentration of all samples was adjusted to 1 μg/ml by dilution with lysis buffer. Samples were processed using the SP3 protocol [53] and on-bead digested overnight at 37 °C with sequencing-grade trypsin (Promega, USA). Peptides were modified with TMTsixplex™ [54] Isobaric Label Reagent (ThermoFisher, USA) according to the manufacturer's instructions. For sample clean up, an OASIS® HLB μElution Plate (Waters Corporation, USA) was used. Offline high-pH reverse phase fractionation was performed on an Agilent 1200 Infinity HPLC system (Agilent Technologies, USA), equipped with a Gemini® C18 column (Phenomenex, USA), resulting in 12 fractions [55] which were further analysed by nano-liquid chromatography coupled to tandem mass spectrometry (nLC-MS/MS). For further details see **Supplementary Information**, Section 3.1.

Quantitative proteomic analysis was performed by nLC-MS/MS. In-line peptide fractionation was performed with an UltiMate™ 3000 RSLC nano system (Dionex Corporation, USA) equipped with an Acclaim™ PepMap™ 100C18 trapping cartridge (ThermoFisher, USA) and a nanoEase™ M/Z HSS T3 C18 analytical column (Waters Corporation, USA). The outlet of the analytical column was coupled directly to an Orbitrap™ Q Exactive™ Plus hybrid quadrupole mass spectrometer (ThermoFisher, USA) using a Nanospray Flex™ Ion Source (ThermoFisher, USA). The peptides were introduced into the mass spectrometer via a Pico-Tip® Emitter and an applied spray voltage of 2.3 kV. Mass spectra were collected in a data-dependent acquisition mode. IsobarQuant [56] and Mascot (v2.2.07) [57] were chosen for data processing. A Uniprot *Homo sapiens* proteome database (UP000005640) containing common contaminants and reversed sequences was used [58]. A mass error tolerance of 10 ppm was set for the full scan (MS1) and 0.02 Da for the MS/MS (MS2) spectra. Trypsin was selected as a protease with an allowance of maximum two missed cleavages. A minimum peptide length of seven amino acids and at least two unique peptides were required for a protein identification. The false discovery rate (FDR) on peptide and protein level was set to 0.01. For further details see **Supplementary Information**, Section 3.2.

### 2.10 Bioinformatics assessment

Data resulting from the quantitative proteomic analysis were processed using R programming language [59]. Only proteins identified with at least two unique peptides in at least two out of the three replicates were retained for analysis. Batch-effects were removed using the respective function from the limma package [60]. The vsn package was used for normalisation [61] and missing values were imputed with the Msnbase package [62]. Differential protein abundance between different conditions was tested with the limma package [60]. In any given comparison, proteins were considered to show significant differences (‘hits’), when a 2-fold change (FC) was detected with an FDR below 0.05. PANTHER (v16.0) Gene List Analysis Tool [63] was used to functionally classify the full proteomic dataset. Protein ‘hits’ were analysed using the Statistical over-representation test from PANTHER (v16.0) Tools [63]. Functional protein association networks were identified using STRING (v11.0) database [64] and the MCL algorithm [65] and further processed with Cytoscape (v3.8.0) [66]. Individual networks were functionally annotated using Reactome (v74) Gene List Analysis Tool [67]. Manual annotation was performed using the Uniprot portal [58] and/or the available scientific literature. For the identification of ECM proteins within the different datasets, the Matrisome Annotator webtool [68] was used. Venn Diagrams were created with the corresponding webtool from the Bioinformatics & Evolutionary Genomics web resource of Dr. Van De Peer Lab [69]. For further details see **Supplementary Information**, Section 3.3.

### 2.11 Statistical assessment

All experiments were performed in triplicate and numerical data are represented as mean values ± standard deviations. SigmaPlot (v12) (Systat Software, Inc, USA) was used for statistical analysis unless otherwise specified. One-way ANOVA followed by Tukey's HSD post-hoc test was used for finding statistically significant differences between the mean values of different groups. Normality and equal variance were tested by means of the Shapiro-Wilk and Levene's tests, respectively. Kruskal-Wallis test was performed when the assumptions of parametric analysis were violated. Unless otherwise specified, the differences between experimental groups were considered statistically significant when calculated p-values were less than 0.05.

## 3 Results

### 3.1 Cell morphology, proliferation, viability and metabolic activity assessment

Qualitative F-actin staining analysis at all time points indicated that hTCs and hDFs possessed a similar spindle-shaped morphology, when cultured under both -MMC and +MMC conditions (Supplementary Fig. S1). Proliferation rate was significantly (p < 0.05) higher in hDFs at all time points and conditions and no significant (p > 0.05) difference was observed between the -MMC and +MMC conditions of a given cell type at a given time point (Supplementary Fig. S2). Cell viability analysis revealed no significant (p > 0.05) differences between hTCs and hDFs when cultured under both -MMC and +MMC conditions (Supplementary Fig. S3). At a given time point, no significant (p > 0.05) difference was observed between the respective hTCs and hDFs groups and the +MMC groups induced significantly (p < 0.05) lower metabolic activity than the -MMC groups (Supplementary Fig. S4).

### 3.2 Gene expression assessment

Gene expression analysis for the -MMC groups (Supplementary Fig. S5) revealed no significant (p > 0.05) differences in expression levels of *COL1A1*, *SOX9* and *RUNX2* between hTCs and hDFs at any time point. *COL3A1* expression was significantly (p < 0.05) higher in hDFs relative to hTCs at day 4, but no significant (p > 0.05) differences were detected at day 7 and day 10. *SCX* and *MKX* expression was significantly (p < 0.05) higher in hTCs relative to hDFs at all time points. *TNMD* expression was too low to be quantified (Supplementary Fig. S6). As a function of time in culture, *COL1A1* expression was significantly (p < 0.05) reduced from day 4 to day 10 in both hTC and hDF cultures; *COL3A1* expression was significantly (p < 0.05) reduced from day 4 to day 10 in hDF cultures; and *SCX* and *MKX* expressions were significantly (p < 0.05) reduced from day 4 to day 7 and day 10 in hTC cultures. Note: Gene expression analysis for the +MMC groups was not conducted due to the inhibitory effects of CR to the reverse transcription reaction (Supplementary Fig. S7).

### 3.3 Immunofluorescent assessment

**Fig. 1:**
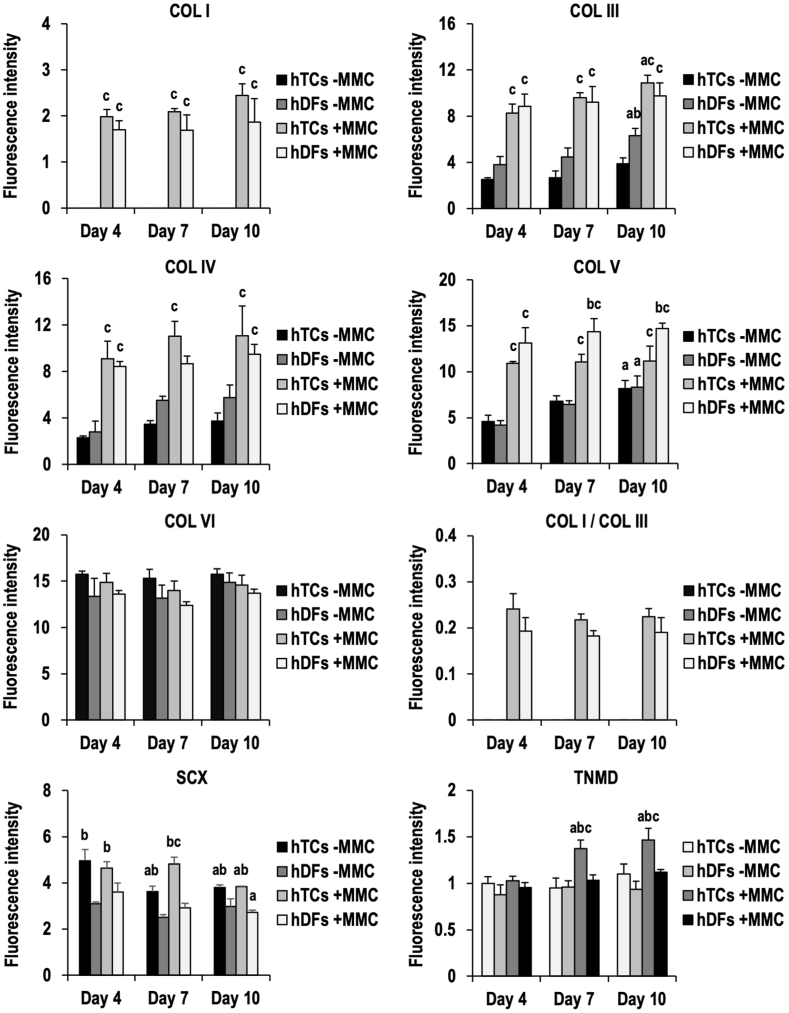
Fluorescence intensity analysis of hTCs and hDFs cultured under -MMC and +MMC conditions. MMC treatment increased the fluorescence intensities of all analysed collagen types in both hTCs and hDFs, relative to control conditions, except for COL IV at day 7 in hDFs, and for COL VI at all time points and conditions. hTCs and hDFs displayed similar fluorescence intensities for all collagen types analysed, except for COL III, which showed significantly higher intensity in hDFs after 10 days of culture under control conditions, and for COL V, which was significantly higher in hDFs after 7 and 10 days of culture under + MMC conditions. Similarly, no significant (p > 0.05) differences were observed between the COL I/COL III ratio of hTCs and hDFs cultured under + MMC conditions. Time in culture increased the deposition (day 10 vs. day 4) of COL III (hDFs -MMC and hTCs + MMC) and COL V (hTCs -MMC and hDFs -MMC). MMC treatment induced higher SCX expression in hTCs after 7 days of culture. hTCs showed higher SCX expression levels than hDFs at all time points and conditions. As a function of time in culture, SCX expression levels decreased after 7 (in hTCs -MMC) and 10 days (in hTCs -MMC, hTCs + MMC, and hDFs + MMC) in culture. MMC treatment increased TNMD expression in hTCs after 7 and 10 days of culture. TNMD Expression was significantly higher in hTCs than in hDFs after 7 and 10 days of culture under + MMC conditions. TNMD expression increased from day 4 to day 7 and day 10 in hTCs + MMC group (a: p < 0.05 vs. the same condition at day 4; b: p < 0.05 between cell types at the same time point and condition; c: p < 0.05 vs. -MMC control at the same time point).

Immunofluorescent analysis (Supplementary Figs. S8–S15) and complementary fluorescence intensity analysis (Fig. 1) revealed that in general, MMC significantly (p < 0.05) increased COL I, III, IV and V deposition at all time points in both hTC and hDF (apart from COL IV at day 7, p > 0.05) cultures. MMC did not significantly (p > 0.05) affect COL VI deposition in both hTC and hDF cultures. Expression of SCX and TNMD was only affected by MMC in hTC cultures, which showed a significantly (p < 0.05) higher fluorescence intensity than the respective -MMC groups at day 7 for SCX and at day 7 and day 10 for TNMD. α-SMA was not detected at any time point and condition. Between hTCs and hDFs, in general, no significant (p > 0.05) differences were observed in COL I, III (apart from day 10, hTCs -MMC significantly, p < 0.05, lower than hDFs -MMC), IV, V (apart from day 7 and day 10, hTCs + MMC significantly, p < 0.05, lower than hDFs + MMC) and VI deposition. Similarly, no significant (p > 0.05) differences were observed between the COL I/COL III ratio of hTCs and hDFs. Expression of SCX was significantly (p < 0.05) higher in hTCs than in hDF cultures at all time points and conditions. TNMD expression was significantly (p < 0.05) higher in hTCs than in hDF cultures at day 7 and day 10 under + MMC conditions. In general, time in culture did not significantly (p > 0.05) affect COL I, III (apart from hDFs -MMC and hTCs + MMC, day 10 significantly, p < 0.05, higher than day 4), IV, V (apart from hTCs -MMC and hDFs -MMC, day 10 significantly, p < 0.05, higher than day 4) and VI deposition, nor the COL I/COL III ratio, in both hTC and hDF cultures. Expression of SCX was significantly (p < 0.05) reduced in hTC cultures from day 4 to day 7 and day 10 in -MMC conditions; from day 4 to day 10 in +MMC conditions; and from day 4 to day 10 in hDF cultures under + MMC conditions. TNMD expression was significantly (p < 0.05) increased from day 4 to day 7 and day 10 in hTC cultures under + MMC conditions.

### 3.4 Collagen deposition via electrophoresis assessment

SDS-PAGE and complementary densitometry analyses (Supplementary Fig. S16) revealed that MMC significantly (p < 0.05) increased COL I deposition in both hTC and hDF cultures at all time points (apart from hDFs at day 4, p > 0.05). Between hTCs and hDFs, only at day 10 in the absence of MMC, the hDFs deposited significantly (p < 0.05) more COL I than the hTCs. As a function of time in culture, COL I deposition was significantly (p < 0.05) increased from day 4 to day 10 in hDFs cultured under -MMC conditions, and in both hTCs and hDFs cultured under + MMC conditions.

### 3.5 Proteolytic activity assessment

Gelatine zymography (Supplementary Fig. S17) revealed the presence of both MMP2 and MMP9 in fresh cell culture medium. No significant (p > 0.05) differences were found in the secretion levels of MMP2 of hTCs and hDFs at any time point and condition. No significant (p > 0.05) differences were found between the levels of MMP9 in fresh and conditioned medium from hTCs and hDFs, at any time point and condition.

### 3.6 Proteomics assessment

A total of 5267 proteins were identified in each group (**Supplementary File S1**), which classified into 22 protein classes, 14 cellular components and 8 molecular functions using PANTHER Gene List Analysis Tool (Supplementary Fig. S18).

#### 3.6.1 hTCs -MMC vs. hDFs -MMC

**Fig. 2:**
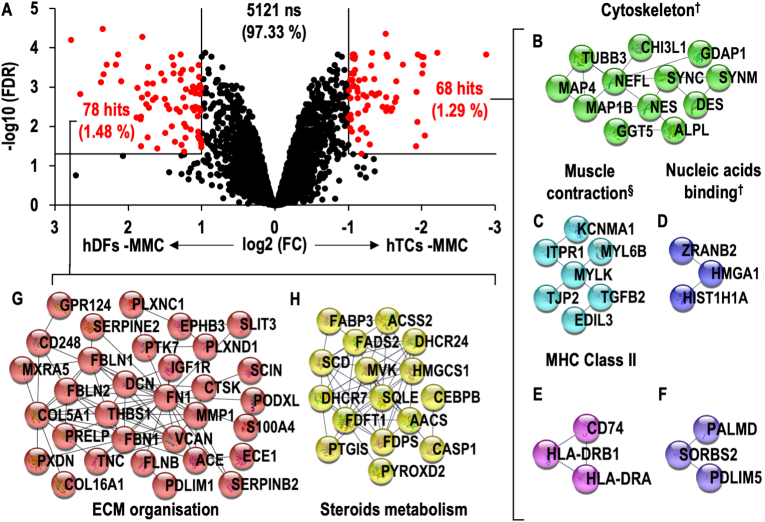
Volcano plot comparing hTCs and hDFs cultured under -MMC conditions **(A)**. Interactomic analysis of proteins found at significantly higher levels in hTCs (**B–F**) and hDFs (**G, H**). Networks were annotated using Reactome (v74) gene list analysis tool unless otherwise stated (^**†**^denotes for manually annotated networks; ^**§**^Haemostasis pathway was also found to be over-represented in the given network).

Among the 5267 identified proteins, 146 proteins (2.77 %) demonstrated significant differences between hTCs and hDFs cultured under -MMC conditions (**Supplementary File S2**). Within them, 68 proteins (1.29 %) were significantly (FC > 2, FDR <0.05) higher in hTCs and 78 (1.48 %) were significantly (FC > 2, FDR <0.05) higher in hDFs (Fig. 2A). PANTHER statistical over-representation tests revealed over-representation of cytoskeletal- and major histocompatibility complex-related proteins within the significantly higher proteins in hTCs. Equally, extracellular region/matrix, as well as lipid biosynthetic proteins were over-represented within the significantly higher proteins in hDFs (Supplementary Table S4). STRING analyses revealed five networks within the significantly higher proteins in hTCs; four of these were annotated as cytoskeleton-, muscle contraction/haemostasis-, nucleic acids binding- and MHC class II antigen presentation-related protein networks (Fig. 2B–E, Supplementary Fig. S19), whilst no annotation was found for the fifth (Fig. 2F). Similar analyses revealed two networks within the significantly higher proteins in hDFs; one related to ECM organisation and one related to steroids metabolism (Fig. 2G–H, Supplementary Figs. S20–S21).

#### 3.6.2 hTCs + MMC vs. hDFs + MMC

**Fig. 3:**
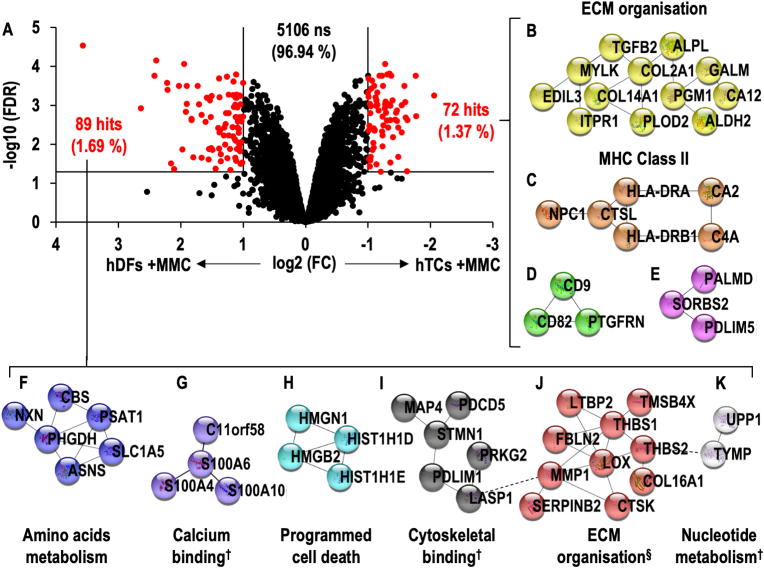
Volcano plot comparing hTCs and hDFs cultured under + MMC conditions **(A)**. Interactomic analysis of proteins found at significantly higher levels in hTCs (**B-E**) and hDFs (**F–K**). Interactions between nodes of networks divided after MCL clustering are represented by dashed lines. Networks were annotated using Reactome gene list analysis tool unless otherwise stated (^**†**^denotes for manually annotated networks; ^**§**^Haemostasis pathway was also found to be over-represented in the given network).

Under + MMC conditions, 161 proteins (3.06 %) demonstrated significant differences between hTCs and hDFs (**Supplementary File S3**) and within them, 72 proteins (1.37 %) were significantly (FC > 2, FDR <0.05) higher in hTCs and 89 (1.69 %) were significantly (FC > 2, FDR <0.05) higher in hDFs (Fig. 3A). Following PANTHER statistical over-representation tests, no over-represented annotations were found within the significantly higher proteins in hTCs, whilst development-, extracellular region/matrix- and calmodulin-related- proteins were over-represented within the significantly higher proteins in hDFs (Supplementary Table S5). STRING analyses revealed four networks within the significantly higher proteins in hTCs; two networks were related to ECM organisation and MHC Class II antigen presentation (Fig. 3B–C, Supplementary Fig. S22), whilst no annotations were found for the remaining two networks (Fig. 3D–E). Similar analyses revealed six networks (amino acids metabolism, calcium binding, programmed cell death, cytoskeletal binding, ECM organisation/haemostasis and nucleotide metabolism) within the significantly higher proteins in hDFs (Fig. 3F–K, Supplementary Figs. S23–S24).

#### 3.6.3 hTCs -MMC vs. hTCs + MMC

**Fig. 4:**
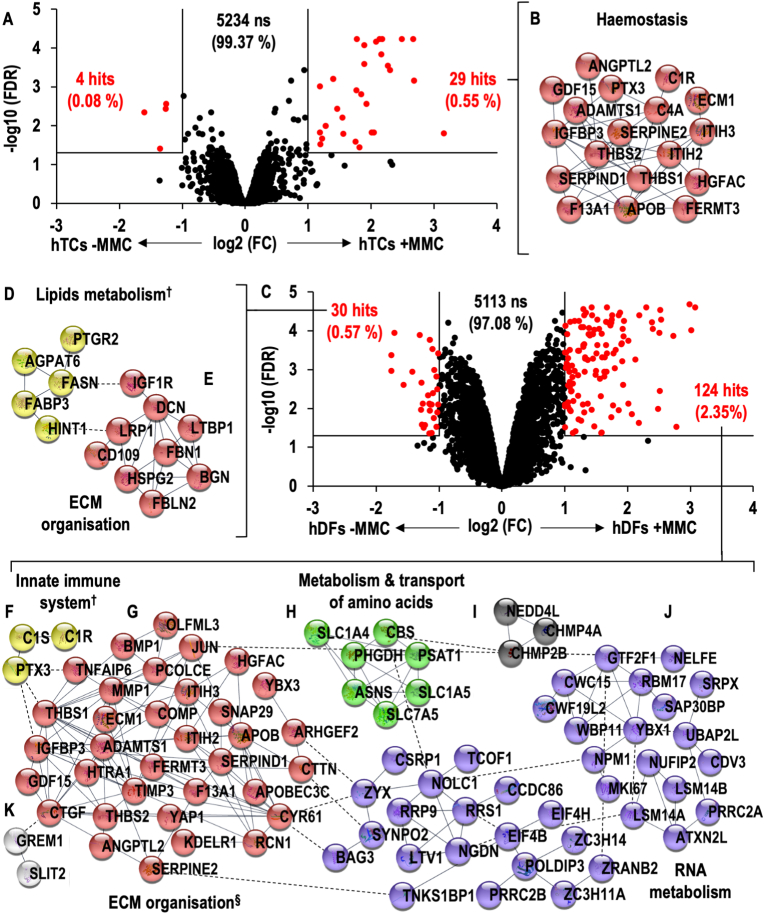
Volcano plot comparing hTCs cultured under -MMC and +MMC conditions (**A**). Interactomic analysis of proteins found at significantly higher levels in hTCs + MMC (**B**). Volcano plot comparing hDFs cultured under -MMC and +MMC conditions (**C**). Interactomic analysis of proteins found at significantly higher levels in hDFs -MMC (**D, E**). Interactomic analysis of proteins found at significantly higher levels in hDFs + MMC (**F–K**). Interactions between nodes of networks divided after MCL clustering are represented by dashed lines. Networks were annotated using Reactome gene list analysis tool unless otherwise stated (^**†**^denotes for manually annotated networks; ^**§**^Haemostasis pathway was also found to be over-represented in the given network).

In hTC cultures, 33 proteins (0.63 %) demonstrated significant differences between -MMC and +MMC conditions (**Supplementary File S4**), of which 4 (0.08 %) and 29 (0.55 %) proteins were significantly (FC > 2, FDR <0.05) higher in -MMC and +MMC cultures, respectively (Fig. 4A). PANTHER statistical over-representation tests revealed no over-represented annotations within the proteins significantly higher in -MMC cultures, whilst proteins related to development, haemostasis, extracellular region/matrix, protease activities and protease inhibitors were over-represented within the significantly higher proteins in +MMC cultures (Supplementary Table S6). STRING analyses demonstrated no interaction networks within the significantly higher proteins in -MMC cultures, whilst one (haemostasis) network was found within the significantly higher proteins in +MMC cultures (Fig. 4B, Supplementary Fig. S25).

#### 3.6.4 hDFs -MMC vs. hDFs + MMC

In hDF cultures, 154 proteins (2.92 %) demonstrated significant differences between -MMC and +MMC conditions (**Supplementary File S5**), of which 30 (0.57 %) and 124 (2.35 %) proteins were significantly (FC > 2, FDR <0.05) higher in -MMC and +MMC cultures, respectively (Fig. 4C). PANTHER statistical over-representation tests revealed over-representation of extracellular matrix proteins within the significantly higher proteins in -MMC cultures, whilst proteins related to extracellular region/matrix, RNA metabolism, RNA splicing, ribosomes, proteases and protease inhibitors were over-represented within the significantly higher proteins in +MMC cultures (Supplementary Table S7). STRING analyses identified two interaction networks within the significantly higher proteins in -MMC cultures, related to lipids metabolism and ECM organisation pathways (Fig. 4D–E, Supplementary Fig. S26). Also, six networks were identified within the significantly higher proteins in +MMC cultures. Four of these were related to the innate immune system, the ECM organisation/haemostasis, the metabolism and transport of amino acids, and the metabolism of RNA (Fig. 4F–J, Supplementary Figs. S27–S28); and no annotations were found for the remaining two networks (Fig. 4I–K).

### 3.7 Matrisome assessment

The Matrisome Annotator webtool revealed a total of 179 ECM proteins within the 5279 proteins identified by means of proteomic analysis. From these, 71 were classified as core matrisome proteins, and 108 as matrisome-associated. The 71 core matrisome proteins were further classified as 13 collagens, 52 glycoproteins and 6 proteoglycans, whilst the 108 matrisome-associated proteins were classified into 59 ECM regulators and 31 ECM affiliated (Supplementary Fig. S29).

#### 3.7.1 hTCs -MMC vs. hDFs -MMC

**Fig. 5:**
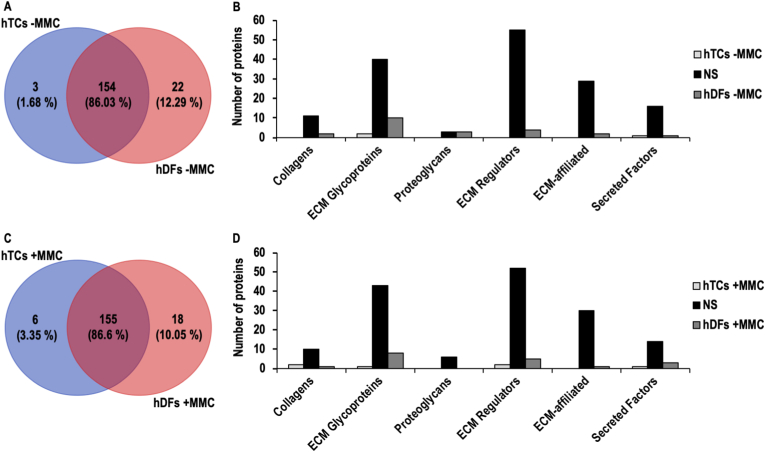
Comparative matrisome analysis of hTCs and hDFs cultured under -MMC and +MMC conditions. Venn diagrams representing the comparison of hTCs and hDFs cultured under -MMC (**A**), and +MMC (**C**) conditions. Classification into matrisome categories of the ECM proteins identified in the comparison of hTCs and hDFs cultured in -MMC (**B**) and +MMC (**D**) conditions. NS denotes for proteins not demonstrating significant differences between the compared groups. hTCs -MMC, and hDFs -MMC, denote for proteins found at significantly higher levels in hTCs or in hDFs, respectively, both under -MMC conditions. hTCs + MMC and hDFs + MMC denote for proteins found at significantly higher levels in hTCs or hDFs, respectively, both under + MMC conditions.

Under -MMC conditions, 154 (86.03 %) ECM proteins did not show significant differences between hTCs and hDFs, whilst 3 (1.68 %) and 22 (12.29 %) ECM proteins were significantly (FC > 2, FDR <0.05) higher in hTCs and hDFs, respectively (Fig. 5A). The 3 significantly higher ECM proteins in hTCs -MMC were classified as 2 glycoproteins and 1 secreted factor and the 18 significantly higher ECM proteins in hDFs -MMC were classified as 2 collagens, 10 glycoproteins, 3 proteoglycans, 4 ECM regulators, 2 ECM affiliated and 1 secreted factor (Fig. 5B–Supplementary Table S8).

#### 3.7.2 hTCs + MMC vs. hDFs + MMC

Under + MMC conditions, 155 (86.6 %) ECM proteins did not show significant differences between hTCs and hDFs, whilst 6 (3.35 %) and 18 (10.05 %) ECM proteins were significantly (FC > 2, FDR <0.05) higher in hTCs and hDFs, respectively (Fig. 5C). The 6 significantly higher ECM proteins in hTCs + MMC were classified as 2 collagens, 1 ECM glycoprotein, 2 ECM regulators, and 1 secreted factor and the 18 significantly higher ECM proteins in hDFs + MMC were classified as 1 collagen, 8 glycoproteins, 5 ECM regulators, 1 ECM affiliated and 3 secreted factors (Fig. 5D–Supplementary Table S9).

#### 3.7.3 hTCs -MMC vs. hTCs + MMC

**Fig. 6:**
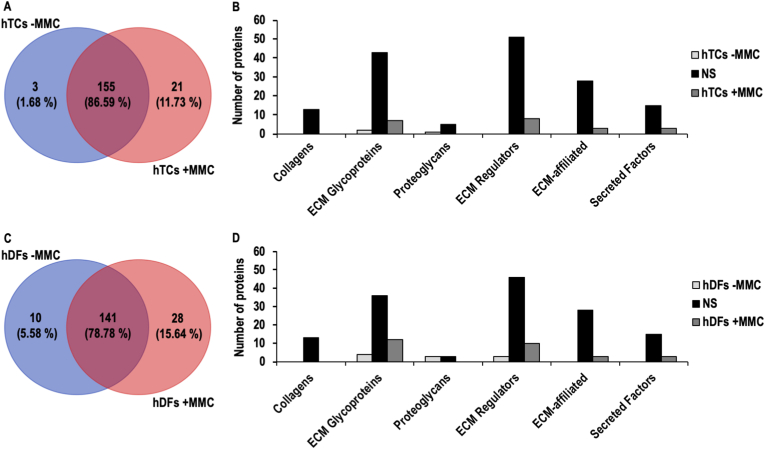
Comparative matrisome analysis of -MMC and +MMC cultures of both hTCs and hDFs. Venn diagrams representing the comparison of the matrisome of hTCs (**A**), and hDFs (**C**), cultured under -MMC and +MMC conditions. Classification into matrisome categories of the ECM proteins identified in the comparison of hTCs (**B**), and hDFs (**D**), cultured under -MMC and +MMC conditions. NS denotes for proteins not demonstrating significant differences between the compared groups. hTCs -MMC, and hDFs -MMC, denote for proteins significantly higher in hTCs, or in hDFs, respectively, both cultured under -MMC conditions. hTCs + MMC and hDFs + MMC denote for proteins significantly higher in hTCs, or in hDFs, respectively, both cultured under + MMC conditions.

In hTC cultures, no significant differences were noted in 155 (86.59 %) ECM proteins and 3 (1.68 %) and 21 (11.73 %) ECM proteins were significantly (FC > 2, FDR <0.05) higher under -MMC and +MMC conditions, respectively (Fig. 6A). The 3 significantly higher ECM proteins in hTCs -MMC were classified as 2 glycoproteins and 1 proteoglycan and the 21 significantly higher ECM proteins in hTCs + MMC were classified as 7 glycoproteins, 8 ECM regulators, 3 ECM affiliated and 3 secreted factors (Fig. 6B–Supplementary Table S10).

#### 3.7.4 hDFs -MMC vs. hDFs + MMC

In hDF cultures, no significant differences were noted for 141 (78.78 %) ECM proteins and 10 (5.58 %) and 28 (15.64 %) ECM proteins were significantly (FC > 2, FDR <0.05) higher under -MMC and +MMC conditions, respectively (Fig. 6C). The 10 significantly higher ECM proteins in hDFs -MMC were classified as 4 glycoproteins, 3 proteoglycans and 3 ECM regulators and the 28 significantly higher ECM proteins in hDFs + MMC were classified as 12 glycoproteins, 10 ECM regulators, 3 ECM affiliated and 3 secreted factors (Fig. 6D–Supplementary Table S11).

## 4 Discussion

In tendon engineering, although the use of both hTCs and hDFs has been advocated, based on positive patient therapeutic outcomes, no product has been developed. It has been argued that financial (i.e. manufacturing costs), as opposed to remedial, reasons prohibit the development of tendon engineering medicines. Considering that MMC has been shown to accelerate the development of functional tissue facsimiles based on the principles of *in vitro* organogenesis, herein an in-depth molecular profiling analysis of hTCs and hDFs was performed under -MMC and +MMC conditions.

### 4.1 Basic cell function analysis

In general, great similarities were found between hTCs and hDFs, both under -MMC and +MMC conditions. Although cell morphology, viability and metabolic activity were similar between hTCs and hDFs, hDFs demonstrated a higher proliferation rate than hTCs under both -MMC and +MMC conditions. Although previous reports have also highlighted this difference [21,22], one should note that several studies have reported for dermal fibroblasts proper integration within tendon tissues in preclinical [70] and clinical [19,20] settings. A slight, but significant reduction in metabolic activity was observed under + MMC for both cell types; results from similar studies using CR as MMC agent range from not detecting significant effects [42,48], to detecting a significant [47] or not significant [37,41] reduction. These variable findings can be explained by a small effect size, which challenges statistical power, as a function of the variability of the experimental system [71]. However, in line with previous studies using CR as MMC agent [39,40,42,48], the analyses of cell morphology, proliferation and viability, discarded any possible cytotoxic effects exerted by CR in +MMC conditions.

### 4.2 Phenotypic analysis

The mRNA levels of *COL1A1* showed no significant differences between hTCs and hDFs and although the mRNA levels of *COL3A1* were initially significantly higher in hDFs than in hTCs, the differences no longer existed at the later time points. Considering that in general both tendon and skin tissues have a similar ratio of collagen type I and collagen type III [72], these results are of no surprise. However, both the arrangement of collagen type I and III fibres [4,73] and the cellular responses to mechanical stimulation [74] differ between tissues, imposing the need to assess mechanical stimulation under assayed conditions. It is worth noting that although we have previously assessed mechanical stimulation and MMC in both cell types, gene expression analysis did not indicate tenogenic differentiation of hDFs, possibly attributed to the short *in vitro* culture periods used [37]. *In situ* though, when DFs were subjected to physiological loads for prolonged periods, they demonstrated safety, efficacy and efficiency in both animal models [70] and human patients [19,20]. hDFs expressed *SCX*, *TNMD* and *MKX*, which is of remarkable importance given their roles in tendon differentiation, maturation, and homeostasis [[75], [76], [77]]. Nonetheless, *SCX* and *MKX* mRNA levels were significantly higher in hTCs than in hDFs at all time points, significantly decreasing in hTCs as a function of culture time. Immunofluorescent analysis of SCX corroborated these findings, demonstrating a similar expression pattern in +MMC cultures. Low expression levels of *TNMD* mRNA were observed in both hTCs and hDFs, which hampered its quantitation due to stochastic amplification effects [78]. These findings are in line with the phenotypic drift phenomenon observed in hTC cultures [26,27] and with the down-regulation of *TNMD* in *SCX*^−/−^ and *MKX*^−/−^ mice [75,77]. However, immunofluorescent analysis of TNMD demonstrated its expression in hTCs and hDFs, as well as its increased expression in hTCs under + MMC conditions. SCX expression was also increased in hTCs as a consequence of +MMC treatment, which is in agreement with previous publications arguing that MMC enhances hTC phenotype [[37], [38], [39], [40]]. Regarding the expression of *trans*-differentiation markers in hTCs and hDFs, no differences were found between the mRNA expression levels of the chondrogenic and osteogenic markers *SOX9* and *RUNX2* [79,80], which are associated heterotopic ossification [81] and the myofibroblast marker α-SMA [82], which is associated with fibrosis [83]. These results are encouraging, as both conditions cause functional disability following tendon repair processes [84,85].

### 4.3 Protein deposition and remodelling analyses

Regarding ECM synthesis, similar capabilities were found between hTCs and hDFs. In general, under -MMC conditions, the content of the collagen types assessed ranged from close to detection limits to very low, whilst + MMC significantly enhanced collagen deposition, in agreement with previous reports (e.g. hTC [[37], [38], [39], [40]] and hDF [[41], [42], [43]] cultures). It is worth noting that under -MMC conditions, a significant collagen type I content was revealed by SDS-PAGE in hDFs after 10 days of culture, which was not confirmed by specific immunostaining; this likely reflects intracellular synthesis, highlighting the importance of ascorbic acid/MMC for extracellular secretion and deposition, respectively. The collagen type I/III ratio, which relates to tenocyte phenotype [26] and influences the mechanical properties of soft tissues [86,87], was similar for both cell types, further enhancing the earlier point that the cells did not undergo myofibroblast differentiation. The secretion of MMP2 and MMP9 proteases that mediate tissue remodelling [88] and impact *in vitro* collagen deposition [42] was similar in every case.

### 4.4 Proteomics analysis

Only a minor fraction (∼3 %) of the proteins identified by nLC-MS/MS demonstrated differential abundance between hTCs and hDFs, both under -MMC and +MMC conditions. These results are surprising, as similar studies comparing the quantitative proteomic profile of different cell types, revealed differences ranging from 15 % to 35 % [[89], [90], [91], [92], [93], [94], [95], [96]].

Under -MMC conditions, muscle contraction/haemostasis pathways were enriched in hTCs, along with cytoskeletal proteins. These results likely reflect the high specialisation of hTCs in mechanosensing [97], as the proteins belonging to such pathways are either mechanoresponsive [[98], [99], [100]] or related to inositol-(1,4,5)-triphosphate and intracellular Ca^2+^ release [101,102], which are central elements in mechanotransduction, together with the cytoskeleton [97,103]. Accordingly, and given the relevance of mechanical stimulation in tendon homeostasis [104], further investigation might be required to ascertain whether these differences can lead to a differential response to mechanical load in hDFs and hTCs. Proteins related to chromatin organisation and RNA splicing [[105], [106], [107]], were also enriched in hTCs, although no specific relation with tendon function was found in the scientific literature. Importantly, the content of HLA-DRA and -DRB1 proteins, the main responsible of acute immune rejection [108], was higher in hTCs than in hDFs. These findings agree with the reported lack of HLA-DR protein expression in hDFs [109] and grants them an advantageous position for allogeneic therapies, in which hDFs have successfully been applied without evidence of clinical rejection [110]. Under -MMC conditions, proteoglycans, integrin- and syndecan-interacting proteins, elastic fibres-forming and interacting proteins and proteins involved in collagen synthesis and degradation were enriched in hDFs. Considering that these components play essential roles in tendon function and homeostasis [104,111,112], these findings can explain the positive outcomes obtained with the application of hDFs in tendon disorders [19,20]. Proteins related to cholesterol biosynthesis and regulation thereof were also enriched in hDFs. Despite hypercholesterolemia relates to tendon xanthomas and increased risk of tendon rupture [113,114], the lipids found within xanthomas derive from circulating low-density lipoproteins [115], making therefore unlikely the development of such conditions, by a locally increased intracellular cholesterol biosynthesis.

Under + MMC conditions, the content of HLA-DR proteins was again higher in hTCs than in hDFs, retaining the latter an advantageous position for allogenic therapies [109,110]. ECM proteins involved in collagen formation, biosynthesis and modification that relate to tendon function [104,111] were enriched in hTCs. Likewise, under + MMC treatment, ECM proteins involved in tendon homeostasis (e.g. collagen turnover and elastic fibres formation processes [104,111,112]) were also enriched in hDFs. Despite in both cases + MMC increased the levels of tendon homeostatic proteins, the related processes somewhat differed, thus long-term studies are needed to ascertain if any specific difference may arise from the application of one or another cell type in tendon regeneration. Proteins whose housekeeping roles improbably can hamper tendon function (e.g. synthesis and transport of amino acids [[116], [117], [118], [119], [120]], catabolism of nucleotides [121,122], regulation of the cytoskeleton [[123], [124], [125], [126]]) were also enriched in hDFs. Also, positive regulators of haemostasis were enriched in hDFs [[127], [128], [129], [130]], which may be an asset regarding their clinical application, especially for coagulopathic patients [131]. Despite certain HMG family and histone H1 protein variants enriched in hDFs were annotated as apoptosis-related, these passively participate in the apoptotic process [132,133], mainly functioning as structural chromatin components [134,135]. Accordingly, and considering their interchangeability in the apoptotic process [133,136], different HMG and H1 histone isotype profiles shall not be related to a different sensitivity to apoptosis, as these typically differ between cell types and cell cycle phases [134,135]. Calcium-binding proteins were also enriched in hDFs under + MMC conditions, some of which were previously identified within the tendon proteome [137,138]. However, only the function of S100A4 has been studied in tendon tissue, where is expressed by resident cells, playing a role in tissue repair [139]. Also, development-related proteins were enriched hDFs, which although speculative at this stage, may reflect the phenotypic plasticity observed in hDFs by some authors [140,141], perhaps facilitating tenogenic differentiation after *in vivo* transplantation.

Proteases targeting ECM components [[142], [143], [144], [145], [146]], protease inhibitors [147], proteases promoting ECM deposition and stability [148,149] and pro-collagen processing proteins BMP1 and PCOLCE [150] were enriched under + MMC conditions in both hTC and hDF cultures; indicating that MMC increases pro-collagen processing, ECM remodelling and ECM stabilisation [[148], [149], [150], [151]]. Considering the high ratio of ECM-to-cells found in tendons and the relevance of its constituent proteins in transmitting mechanical forces and maintaining an adequate microenvironment for its resident cells [7,112], these results underscore the potential of MMC in tendon engineering. The HGF-activating protease HGFAC [152] was also enriched in +MMC groups, which, given the role played by HGF in tissue repair and regeneration [153], may be an asset in regenerative settings. Proteins involved in the fibrin clot formation [[154], [155], [156]], fibrinogenesis and fibrinolysis regulators [157,158] were enriched in +MMC cultures; this may improve tissue engineered construct engraftment after surgical implantation [159], whilst balancing the haemostatic process against thrombosis [158,160]. Proteins from the complement system [161,162] and the complement-interacting protein PTX3 [163] were also enriched under + MMC conditions; considering their antimicrobial functions [[161], [162], [163]] and the tissue reparative effects of PTX3 [164], the clinical application of +MMC may be associated to lower post-operative infection rates and better healing profiles. In agreement with the instructive roles of the ECM during development [165], development-related ECM proteins were enriched in hTC cultures as a result of +MMC treatment. Remarkably, these proteins possess antiangiogenic properties [[166], [167], [168], [169]], which likely help to maintain the hypovascular condition of tendon tissue. Proteins involved in RNA maturation and translation [[170], [171], [172], [173]] and amino acid biosynthetic enzymes and transporters [[116], [117], [118], [119], [120],^174^] were enriched in hDFs under + MMC conditions, pointing towards an increased overall protein synthesis rate [175], which is the hallmark of cell and tissue growth [176]. Interestingly, such cellular responses are mainly induced by growth factors [177], whose contents are increased in the ECM of cultured cells by + MMC treatment [178]. It is worth noting that the content of certain proteins (e.g. proteoglycans, elastic fibres-associated proteins [104,112], proteins involved in lipids metabolism [[179], [180], [181], [182]]) was decreased in hDFs by + MMC treatment, whilst this effect was almost negligible in hTC cultures. Intriguingly, whilst under -MMC conditions, the content of these proteins was higher in hDFs than in hTCs, under + MMC conditions such differences no longer existed; MMC therefore may help balancing the levels of certain proteins between cultures of both cell types.

### 4.5 Matrisome analysis

From the 179 ECM proteins identified by matrisome analysis, the ∼86 % demonstrated no differences between hTCs and hDFs. Only 3 (∼1.5 %) and 6 (∼3.5 %) matrisomal proteins were enriched in -MMC and +MMC hTCs, respectively, whilst the fraction of matrisomal proteins enriched in hDFs under both experimental conditions ranged from 10 to 12 %. Among the few matrisomal proteins enriched in hTCs, tendon related proteins (e.g. collagen types II and collagen type XIV [183,184], procollagen lysyl hydroxylase PLOD2 [185,186], growth factor TGFB2 [187,188]) and proteins with diverse functions (e.g. procoagulant protein FGL2 [189], protease CTSL [190], integrin-interacting protein EDIL3 [191]) were found. Apart from the cell dissociation and repulsion receptors Plexin-C1 and -D1 [192] and the inhibitor of fibrinolysis SERPINB2 [193], the vast majority of the matrisome proteins enriched in hDFs have been previously identified in healthy tendons [137,138,183,186,[194], [195], [196]]. This provides an excellent explanation for the reported preclinical and clinical success of hDFs in tendon regeneration [19,20,70,197,198] and highlights the potential of hDFs in tendon engineering. In addition, given the relatively high levels of TGFB2 found in hTCs and considering its critical role during tendon development [187,188], this molecule emerges as a potential tool to shift the phenotype of hDFs towards tenogenic lineage.

Significant overlap was observed between the matrisomal proteins modulated by + MMC in both cell types and, interestingly, cell type specific regulation occurred for only a fraction of their matrisome. Although the functions of a number of these proteins were ascertained during whole-proteome analysis [[142], [143], [144], [145], [146],[148], [149], [150], [151], [152], [153], [154], [155], [156], [157], [158], [159], [160],^166^,^168^,^169^,^199^], additional ECM proteins were annotated by matrisome analysis. Remarkably, most of the proteins enriched in +MMC cultures were components of the proteome of healthy tendons [137,138,183,196,[200], [201], [202]], highlighting the benefits of applying MMC in tendon engineering, using either hTCs or hDFs. CTGF and CYR61 were specifically enriched in hDFs under + MMC, which, given the tenogenic properties of CTGF [203] and the functional *in vitro* equivalence of both proteins [204], may be an asset in tendon engineering. Matrisome proteins with varied functions (e.g. CRISPLD2, TNFAIP6, GREM1, which anti-inflammatory, anti-fibrotic and anti-osteogenic properties [[205], [206], [207]] may be beneficial in tendon engineering) were also enriched in +MMC cultures. Enrichment of collagen proteins under + MMC conditions was not detected by nLC-MS/MS; this may be attributed to the in-solution nature of this technique [[53], [54], [55]] and the poor solubility of collagen [68,201,208]. The levels of certain matrisomal proteins were also reduced in +MMC cultures, which in the case of hDFs, diminished the differences with hTCs, in terms of tendon-related and tendon-unrelated proteins. Only the integrin-interacting protein EDIL3 [191] was specifically enriched in cultures of hTCs, although no relation with tendon function was found in the scientific literature. Interestingly, +MMC diminished FBLN2 and HSPG2 levels in both cell types, both of which are up-regulated during early stages of wound healing [[209], [210], [211], [212]]. This may be driven by the ECM-rich microenvironment produced by MMC, possibly reverting the early-wound transcriptional program induced through cell culturing [213] and subjecting the cells to a more physiological microenvironment *in vitro* [178,214].

## 5 Conclusions

Despite the fact that hDFs and hTCs are used extensively in tendon engineering, the protracted *in vitro* culture required to develop a tissue engineered medicine and the associated exorbitant manufacturing costs have prohibited commercialisation. Considering the MMC can substantially expedite the development of tissue engineered devices, herein an in-depth analysis of hDFs and hTCs under standard and MMC conditions was conducted. This works firstly highlights that hDFs have very high similarity to hTCs, a fact that makes them an excellent cell candidate for tendon engineering. We also found that MMC not only increases and accelerates ECM deposition but also, by doing so, enhances haemostatic, anti-microbial and tissue-protective activities. Thus, this work further de-risks the MMC technology for the accelerated development of advanced therapy medicinal products. Although in skin wound healing MMC-derived scaffold-free constructs have shown promise [44], in tendon engineering we believe that this technology can be used in conjugation with a carrier system that will provide appropriate mechanical resilience.

## CRediT authorship contribution statement

**Adrian Djalali-Cuevas:** Data curation, Formal analysis, Investigation, Methodology, Writing – original draft, Writing – review & editing. **Mandy Rettel:** Formal analysis, Methodology. **Frank Stein:** Formal analysis, Methodology. **Mikhail Savitski:** Formal analysis, Methodology. **Stephen Kearns:** Resources. **Jack Kelly:** Resources. **Manus Biggs:** Writing – original draft. **Ioannis Skoufos:** Project administration, Resources, Supervision. **Athina Tzora:** Project administration, Resources, Supervision. **Nikitas Prassinos:** Project administration, Supervision. **Nikolaos Diakakis:** Project administration, Supervision. **Dimitrios I. Zeugolis:** Conceptualization, Funding acquisition, Investigation, Methodology, Project administration, Resources, Supervision, Visualization, Writing – original draft, Writing – review & editing.

## Declaration of competing interest

The authors declare that they have no known competing financial interests or personal relationships that could have appeared to influence the work reported in this paper.

## Data Availability

Data will be made available on request.
