# Supplementary material for: Macromolecular crowding in human tenocyte and skin fibroblast cultures: A comparative analysis
Source: Mater Today Bio. 2024 Jan 28;25:100977. doi: 10.1016/j.mtbio.2024.100977 (PMC10846491; doi:10.1016/j.mtbio.2024.100977)
Supplement: Multimedia component 1 [file mmc1.docx]

**Supplementary Information**

# Title

Macromolecular crowding in human tenocyte and skin fibroblast cultures: A comparative analysis

# Authors

Adrian Djalali-Cuevas (1, 2, 3), Mandy Rettel (4), Frank Stein (4), Mikhail Savitski (4), Stephen Kearns (5), Jack Kelly (6), Manus Biggs (7), Ioannis Skoufos (1), Athina Tzora (1), Nikitas Prassinos (2), Nikolaos Diakakis (2), Dimitrios I. Zeugolis* (3)

# Affiliations

1. Laboratory of Animal Science, Nutrition and Biotechnology, School of Agriculture, University of Ioannina, Arta, Greece

2. School of Veterinary Medicine, Aristotle University of Thessaloniki, Thessaloniki, Greece

3. Regenerative, Modular & Developmental Engineering Laboratory (REMODEL), Charles Institute of Dermatology, Conway Institute of Biomolecular & Biomedical Research and School of Mechanical & Materials Engineering, University College Dublin (UCD), Dublin, Ireland

4. Proteomics Core Facility, European Molecular Biology Laboratory (EMBL), Heidelberg, Germany

5. Merlin Park University Hospital, Galway, Ireland

6. Galway University Hospital, Galway, Ireland

7. Science Foundation Ireland (SFI) Centre for Research in Medical Devices (CÚRAM), Biomedical Sciences Building, University of Galway, Galway, Ireland

* Corresponding Author: Dimitrios I. Zeugolis, REMODEL, UCD, Ireland. Telephone: +353 17 16 18 84; Email: dimitrios.zevgolis@ucd.ie

# 1. Supplementary Figures

**Supplementary Figure S1:** Analysis of cell morphology of hTCs and hDFs cultured under -MMC and +MMC conditions. Both cell types possessed a similar spindle-shaped morphology at all experimental time-points and conditions. Treatment with MMC did not affect the morphology of hTCs nor hDFs at any analysed time point relative to hTCs and hDFs cultured under -MMC conditions.

**Supplementary Figure S2:** Assessment of cell proliferation of hTCs and hDFs cultured under -MMC and +MMC conditions, by means of nuclear fluorescent staining (**A**) and complementary image analysis (**B**). Proliferation was higher in hDFs than in hTCs, both in the absence and presence of MMC. MMC did not affect the proliferation rate of any of the two cell types. (a: p < 0.05 vs. the same condition at day 4; b: p < 0.05 between cell types at the same time point and condition).

**Supplementary Figure S3:** Qualitative (A) and quantitative (B) viability assessment of hTCs and hDFs cultured under -MMC and +MMC conditions. No significant differences (p > 0.05) were detected as a function of cell type, absence and presence of MMC and time point.

**Supplementary Figure S4:** Assessment of cell metabolic activity of hTCs and hDFs cultured under -MMC or +MMC conditions. No statistically significant differences were found between the metabolic activity of hTCs and hDFs under any experimental conditions or at any experimental time-points. MMC treatment slightly reduced the metabolic activity in both cell types in a similar fashion. (c: p < 0.05 vs respective -MMC group at the same time point).

**Supplementary Figure S5:** Analysis of the expression of tendon-, cartilage-, and bone- related genes in hTCs and hDFs. *COL1A1* mRNA expression levels were similar between hTCs and hDFs and decreased over the course of the experiment. No statistically significant differences were detected for *SOX9* and *RUNX2*. *COL3A1* mRNA expression levels were initially higher in hDFs than in hTCs but showed no statistically significant difference with the levels of hTCs at day 10. *SCX* and *MKX* mRNA expression was higher in hTCs than in hDFs at all time points, although the expression levels of both genes decreased in hTCs after day 4. (a: p < 0.05 vs. the same condition at day 4; b: p < 0.05 between cell types at the same time point).

**Supplementary Figure S6:** RT-qPCR amplification curves (**A**) and melting curve analysis (**B**) of representative samples of hTCs and hDFs evaluated for *TNMD* expression. Although low expression levels (Ct ~ 35) and stochastic effects were observed during amplification (e.g. high variability or stochastic amplification fails between technical replicates), *TNMD* expression was detected in both hTCs and hDFs at all experimental time points. Melting curve analysis demonstrated the amplification of a single PCR product with a melting temperature corresponding with the estimated values of the target amplicon. (d4, d7 and d10 denote for the different analysed time-points; A and B denote for technical replicates).

**Supplementary Figure S7:** Assessment of RT-qPCR inhibition by MMC. (**A**) Experimental design. (**B**) Cycle threshold (Ct) values vs. log10 cDNA of the serial dilutions of RNA isolated from the three different assessed conditions (-MMC: control cultures; -MMC+: control cultures with spiked-in MMC into the RNA isolation reagent; +MMC: MMC-treated cultures). Inhibition of RT-qPCR by MMC is appreciated due to non-linear relation of Ct vs Log10 cDNA in +MMC group, in contrast with the linearity seen in the -MMC group. The inability of the RNA isolation protocol to efficiently remove the MMC from the RNA samples, and the consequent inhibition of the RT-qPCR, can be appreciated due to an increase of the Ct values of the -MMC+ group when compared with the -MMC group, as well as its non-linear relationship between Ct and log10 cDNA.

**Supplementary Figure S8:** Fluorescent immunostaining for collagen type I in hTCs and hDFs cultured under -MMC and +MMC conditions. In the absence of MMC, levels of collagen type I fibres were close to detection limit in cultures of both cell types. Deposition of collagen type I fibres dramatically increased with MMC treatment in both types of cells. When treated with MMC, hTCs and hDFs contained similar amounts of collagen type I fibres.

**Supplementary Figure S9:** Fluorescent immunostaining for collagen type III in hTCs and hDFs cultured under -MMC and +MMC conditions. In the absence of MMC, the amounts of collagen type III fibres were slightly higher in hDFs than in hTCs, especially after 10 days of culture. Deposition of collagen type III fibres dramatically increased with MMC treatment in both types of cells. When treated with MMC, hTCs and hDFs contained similar amounts of collagen type III fibres.

**Supplementary Figure S10:** Fluorescent immunostaining for collagen type IV in hTCs and hDFs cultured under -MMC and +MMC conditions. Similar amounts of fibres of collagen type IV were deposited in hTCs and hDFs under -MMC and +MMC conditions. When treated with MMC, the deposition of collagen type IV fibres greatly increased in both types of cells.

**Supplementary Figure S11:** Immunofluorescent staining for collagen type V in hTCs and hDFs cultured under -MMC and +MMC conditions. In the absence of MMC, deposition of collagen type V fibres was similar between hTCs and hDFs. MMC increased the deposition of collagen type V fibres in both types of cells. When treated with MMC, comparable amounts of collagen type V fibres were deposited in both hTCs and hDFs, although slightly higher amounts were found in hDFs at the last time points.

**Supplementary Figure S12:** Immunofluorescent staining for collagen type VI in hTCs and hDFs cultured under -MMC and +MMC conditions. In the absence of MMC, deposition of collagen type VI fibres was similar in both hTCs and hDFs. MMC did not increase the deposition of collagen type VI fibres in any case.

**Supplementary Figure S13:** Fluorescent immunostaining for SCX in hTCs and hDFs cultured under -MMC and +MMC conditions. +MMC culture conditions significantly increased the staining intensity of SCX in hTCs at day 7. SCX staining intensity was higher in hTCs than in hDFs at all time points and conditions. SCX staining intensity decreased in hTCs after 7 and 10 days of culture under -MMC conditions, and in hTCs and hDFs after 10 days of culture under +MMC conditions.

**Supplementary Figure S14:** Fluorescent immunostaining for TNMD in hTCs and hDFs cultured under -MMC and +MMC conditions. +MMC conditions increased TNMD staining intensity in hTCs at day 7 and day 10. TNMD staining intensity was higher in hTCs than in hDFs at day 7 and day 10 under +MMC conditions. TNMD staining intensity was increased in hTCs from day 4 to day 7 and day 10 under +MMC conditions.

**Supplementary Figure S15:** Fluorescent immunostaining of α-SMA in hTCs and hDFs cultured under -MMC and +MMC conditions. α-SMA was not detected under any experimental conditions at any experimental time-points (**+**: positive control).

**Supplementary Figure S16:** SDS-PAGE collagen content analysis of hTCs and hDFs cultured under -MMC and +MMC conditions (**A**), and corresponding densitometric analysis (**B**). +MMC conditions significantly increased collagen type I content in both hTCs and hDFs. No statistically significant differences were found between the collagen content of hTCs and hDFs cultured under +MMC conditions. Under -MMC conditions, significant levels of collagen type I were only detected in hDFs after 10 days of culture. (STD: Symatese pepsin-extracted purified bovine type I collagen; *α*1: type I collagen alpha I chain; *α*2: type I collagen alpha 2 chain; *β*1: *α*1*α*1 dimer; *β*2: *α*1*α*2 dimer; *γ*: *α*1*α*1*α*2 trimer; a: p < 0.05 vs. the same condition at day 4; b: p < 0.05 between cell types at the same time point and condition; c: p < 0.05 vs. respective -MMC group at the same time point).

**Supplementary Figure S17:** Secretion of MMPs by hTCs and hDFs cultured under -MMC and +MMC conditions analysed by gelatine zymography (**A**). Densitometric analysis revealed no significant differences (p > 0.05) in the MMP2 secretion levels of hTCs and hDFs at any time point and condition (**B**). The levels of MMP9 in conditioned medium of hTCs and hDFs were similar to the levels detected in fresh medium (**C**). (C-: fresh cell culture medium).

**Supplementary Figure S18:** Functional classification of proteins identified by nLC-MS/MS, using PANTHER classification system. A large number of different protein classes were identified within the proteomic dataset. These proteins belonged to a variety of cellular components and carry out a number of different molecular functions.

**Supplementary Figure S19:** Reactome pathway analysis of interaction networks identified within the proteins found at significantly higher levels in hTCs, following quantitative proteomic comparison of hTCs and hDFs under -MMC conditions. (**A, B**). Over-represented pathways in the network of interacting proteins shown in **Figure 2C**. (**C**). Over-represented pathways in the network of interacting proteins shown in **Figure 2E**. Pathways and sub-pathways significantly over-represented in the different analysed networks are framed in orange colour. The colour contained in each frame represents the non-corrected p-value for the corresponding pathway, resulting from the over-representation analysis, according to the colour scale.

**Supplementary Figure S20:** Reactome pathway analysis of interaction networks identified within the proteins found at significantly higher levels in hDFs, following quantitative proteomic comparison of hTCs and hDFs cultured under -MMC conditions. (**A**). Over-represented pathways in the network of interacting proteins shown in **Figure 2G**. (**B – E**). Detailed view of sub-pathways enriched in the network shown in **Figure 2G**. Pathways and sub-pathways significantly over-represented in the different analysed networks are framed in orange colour. The colour contained in each frame represents the non-corrected p-value for the corresponding pathway, resulting from the over-representation analysis, according to the colour scale.

**Supplementary Figure S21:** Reactome pathway analysis of interaction networks identified within the proteins found at significantly higher levels in hDFs, following quantitative proteomic comparison of hTCs and hDFs cultured under -MMC conditions. (**A**). Over-represented pathways in the network of interacting proteins shown in **Figure 2H**. Pathways and sub-pathways significantly over-represented in the different analysed networks are framed in orange colour. The colour contained in each frame represents the non-corrected p-value for the corresponding pathway, resulting from the over-representation analysis, according to the colour scale.

**Supplementary Figure S22:** Reactome pathway analysis of interaction networks identified within proteins found at significantly higher levels in hTCs, following quantitative proteomic comparison of hTCs and hDFs cultured under +MMC conditions. (**A**). Over-represented pathways in the network of interacting proteins shown in **Figure 3B**. (**B**). Detailed view of over-represented sub-pathways in the network of interacting proteins shown in **Figure 3B**. (**C**). Over-represented pathways in the network of interacting proteins shown in **Figure 3C**. Pathways and sub-pathways significantly over-represented in the different analysed networks are framed in orange colour. The colour contained in each frame represents the non-corrected p-value for the corresponding pathway, resulting from the over-representation analysis, according to the colour scale.

**Supplementary Figure S23:** Reactome pathway analysis of interaction networks identified within proteins found at significantly higher levels in hDFs, following quantitative proteomic comparison of hTCs and hDFs cultured under +MMC conditions. (**A**). Over-represented pathways in the network of interacting proteins shown in **Figure 3F**. (**B**). Over-represented pathways in the network of interacting proteins shown in **Figure 3H**. (**C**). Over-represented pathways in the network of interacting proteins shown in **Figure 3J**. (**D**). Detailed view of over-represented sub-pathways in the network of interacting proteins shown in **Figure 3H**. Pathways and sub-pathways significantly over-represented in the different analysed networks are framed in orange colour. The colour contained in each frame represents the non-corrected p-value for the corresponding pathway, resulting from the over-representation analysis, according to the colour scale.

**Supplementary Figure S24:** Reactome pathway analysis of interaction networks identified within proteins found at significantly higher levels in hDFs, following quantitative proteomic comparison of hTCs and hDFs cultured under +MMC conditions. (**A**). Over-represented pathways in the network of interacting proteins shown in **Figure 3J**. (**B, C**). Detailed view of over-represented sub-pathways in the network of interacting proteins shown in **Figure 3J**. Pathways and sub-pathways significantly over-represented in the different analysed networks are framed in orange colour. The colour contained in each frame represents the non-corrected p-value for the corresponding pathway, resulting from the over-representation analysis, according to the colour scale.

**Supplementary Figure S25:** Reactome pathway analysis of interaction networks identified within proteins found at significantly higher levels in hTCs cultured under +MMC conditions, following quantitative proteomic comparison of hTCs cultured under -MMC and +MMC conditions. (**A**). Over-represented pathways in the network of interacting proteins shown in **Figure 4B**. (**B, C**). Detailed view of over-represented sub-pathways in the network of interacting proteins shown in **Figure 4B**. Pathways and sub-pathways significantly over-represented in the different analysed networks are framed in orange colour. The colour contained in each frame represents the non-corrected p-value for the corresponding pathway, resulting from the over-representation analysis, according to the colour scale.

**Supplementary Figure S26:** Reactome pathway analysis of interaction networks identified within proteins found at significantly higher levels in hDFs cultured under -MMC conditions, following quantitative proteomic comparison of hDFs cultured under -MMC and +MMC conditions. (**A**). Over-represented pathways in the network of interacting proteins shown in **Figure 4E**. (**B**). Detailed view of over-represented sub-pathways in the network of interacting proteins shown in **Figure 4E**. Pathways and sub-pathways significantly over-represented in the different analysed networks are framed in orange colour. The colour contained in each frame represents the non-corrected p-value for the corresponding pathway, resulting from the over-representation analysis, according to the colour scale.

**Supplementary Figure S27:** Reactome pathway analysis of interaction networks identified within proteins found at significantly higher levels in hDFs cultured under -MMC conditions, following quantitative proteomic comparison of hDFs cultured under -MMC and +MMC conditions. (**A, D**). Over-represented pathways in the network of interacting proteins shown in **Figure 4G**. (**B, C, E, F**). Detailed view of over-represented sub-pathways in the network of interacting proteins shown in **Figure 4G**. Pathways and sub-pathways significantly over-represented in the different analysed networks are framed in orange colour. The colour contained in each frame represents the non-corrected p-value for the corresponding pathway, resulting from the over-representation analysis, according to the colour scale.

**Supplementary Figure S28:** Reactome pathway analysis of interaction networks identified within proteins found at significantly higher levels in hDFs cultured under +MMC conditions, following quantitative proteomic comparison of hDFs cultured under -MMC and +MMC conditions. (**A, B**) Over-represented pathways in the network of interacting proteins shown in **Figure 4H**. (**D**) Over-represented pathways in the network of interacting proteins shown in **Figure 4J**. (**C**) Detailed view of sub-pathways over-represented in the network of interacting proteins shown in **Figure 4H**. (**E**) Detailed view of sub-pathways over-represented in the network of interacting proteins shown in **Figure 4J**. Pathways and sub-pathways significantly over-represented in the different analysed networks are framed in orange colour. The colour contained in each frame represents the non-corrected p-value for the corresponding pathway, resulting from the over-representation analysis, according to the colour scale.

**Supplementary Figure S29:** Annotation of ECM proteins within the 5,267 proteins identified by means of proteomic analysis, according to matrisome divisions and categories, using the Matrisome Annotator webtool. A total of 179 ECM proteins were identified, from which 71 were annotated as core matrisome, and 108 were annotated as matrisome-associated (**A**). From the 71 proteins annotated as core matrisome, 13 were annotated as collagens, 52 as ECM glycoproteins and 6 as proteoglycans. From the 108 proteins annotated as matrisome-associated, 59 were annotated as ECM regulators, 31 as ECM-affiliated, and 18 as secreted factors (**B**).

# 2. Supplementary Tables

| **Supplementary Table S1:** Patient details | | | |
| --- | --- | --- | --- |
| **Cell type** | **Sex** | **Age** | **Race** |
| hTCs | Male | 23 | Caucasian |
| hTCs | Male | 18 | Caucasian |
| hTCs | Female | 17 | Caucasian |
| hDFs | Female | 38 | Caucasian |
| hDFs | Male | 28 | African American |
| hDFs | Female | 40 | Asian |

| **Supplementary Table S2:** RT-qPCR primer sequences and characteristics. | | | | | | |
| --- | --- | --- | --- | --- | --- | --- |
| **Gene** | **Primers** | **Tm (ºC)** | **GC (%)** | **Product length** | **PCR efficiency** | **r^2^** |
| COL1A1 | (F) 5´-CAAGAGGAAGGCCAAGTCGAG-3´ | 60.7 | 57.1 | 78 bp | 103.5% | 0.99 |
|  | (R) 5´-CATGGTACCTGAGGCCGTTC-3´ | 60.5 | 60.0 |  |  |  |
| COL3A1 | (F) 5´-TACTTCTCGCTCTGCTTCATCC-3´ | 59.9 | 50.0 | 81 bp | 106.2% | 0.99 |
|  | (R) 5´-ACTGACCAAGATGGGAACATCC-3´ | 60.3 | 50.0 |  |  |  |
| SCX | (F) 5´-GATCTGCACCTTCTGCCTCAG-3´ | 60.7 | 57.1 | 72 bp | 99.7% | 0.99 |
|  | (R) 5´-CTGCGAATCGCTGTCTTTCTG-3´ | 59.9 | 52.4 |  |  |  |
| TNMD | (F) 5´-GGCATCTACTTCGTGGGTCTTC-3´ | 60.7 | 54.6 | 142 bp | N/A | N/A |
|  | (R) 5´-GGACCCAAATCACTGACTGTTC-3´ | 59.2 | 50.0 |  |  |  |
| MKX | (F) 5´-GTCTCCATCGTCATCAGAAACTG-3´ | 59.1 | 47.8 | 96 bp | 98.7% | 0.99 |
|  | (R) 5´-TTTCTGTTAGCTGCGCTTTCAC-3´ | 60.0 | 45.5 |  |  |  |
| SOX9 | (F) 5´-TACCCGCACTTGCACAACG-3´ | 61.3 | 57.9 | 73 bp | 99.8% | 0.99 |
|  | (R) 5´-TCTCGCTCTCGTTCAGAAGTC-3´ | 59.5 | 52.4 |  |  |  |
| RUNX2 | (F) 5´-TGCACCAAGTCCTTTTAATCCAC-3´ | 59.4 | 43.5 | 107 bp | 104.7% | 0.99 |
|  | (R) 5´-TCAGGTAGGAGGGGTAAGACTG-3´ | 60.0 | 54.6 |  |  |  |

| **Supplementary Table S3:** List of antibodies used and their characteristics | | | | | |
| --- | --- | --- | --- | --- | --- |
| **Antigen detected** | **Host species** | **Clonality** | **Dilution factor** | **Product number** | **Manufacturer** |
| SCX | Rabbit | Polyclonal | 1:100 | ab58655 | Abcam, United Kingdom |
| TNMD | Rabbit | Polyclonal | 1:200 | ab203676 | Abcam, United Kingdom |
| aSMA | Mouse | Monoclonal | 1:400 | A2547 | Sigma-Aldrich, Germany |
| Collagen type I | Mouse | Monoclonal | 1:200 | ab90395 | Abcam, United Kingdom |
| Collagen type III | Rabbit | Polyclonal | 1:200 | ab7778 | Abcam, United Kingdom |
| Collagen type IV | Rabbit | Polyclonal | 1:200 | ab6586 | Abcam, United Kingdom |
| Collagen type V | Rabbit | Polyclonal | 1:200 | ab7046 | Abcam, United Kingdom |
| Collagen type VI | Rabbit | Polyclonal | 1:200 | ab6588 | Abcam, United Kingdom |
| Mouse IgG | Goat | Polyclonal | 1:400 | A-32727 | Invitrogen, Ireland |
| Rabbit IgG | Goat | Polyclonal | 1:400 | A-32731 | Invitrogen, Ireland |

| **Supplementary Table S4:** PANTHER statistical over-representation tests for protein hits in hTCs -MMC vs. hDFs -MMC. | | | | |
| --- | --- | --- | --- | --- |
| **Category** | **Sub-category** | **p-value** | **FDR** | **Enriched in** |
| Cellular Component | Cytoskeleton (GO:0005856) | 4.95E-04 | 3.59E-02 | hTCs -MMC |
|  | Supramolecular complex (GO:0099080) | 4.17E-05 | 7.07E-03 | hTCs -MMC |
|  | Supramolecular polymer (GO:0099081) | 4.05E-05 | 1.03E-02 | hTCs -MMC |
|  | Supramolecular fibre (GO:0099512) | 3.47E-05 | 1.76E-02 | hTCs -MMC |
|  | Polymeric cytoskeletal fibre (GO:0099513) | 9.53E-05 | 8.07E-03 | hTCs -MMC |
|  | Actin filament bundle (GO:0032432) | 6.78E-04 | 4.31E-02 | hTCs -MMC |
|  | Actomyosin (GO:0042641) | 8.47E-05 | 8.60E-03 | hTCs -MMC |
|  | Stress fibre (GO:0001725) | 7.47E-05 | 9.48E-03 | hTCs -MMC |
| Protein Class | Major histocompatibility complex protein (PC00149) | 4.65E-04 | 4.49E-02 | hTCs -MMC |
|  | Cytoskeletal protein (PC00085) | 4.52E-04 | 8.72E-02 | hTCs -MMC |
|  |  |  |  |  |
| Biological Process | Lipid biosynthetic process (GO:0008610) | 8.80E-06 | 1.92E-02 | hDFs -MMC |
| Cellular Component | Extracellular region (GO:0005576) | 2.80E-06 | 1.42E-03 | hDFs -MMC |
|  | Extracellular space (GO:0005615) | 1.84E-04 | 3.12E-02 | hDFs -MMC |
|  | Extracellular matrix (GO:0031012) | 4.80E-06 | 1.22E-03 | hDFs -MMC |
| Protein Class | Extracellular matrix protein (PC00102) | 4.48E-05 | 8.65E-03 | hDFs -MMC |
|  | Extracellular matrix structural protein (PC00103) | 2.27E-04 | 2.19E-02 | hDFs -MMC |

| **Supplementary Table S5:** PANTHER statistical over-representation tests for protein hits in hTCs +MMC vs. hDFs +MMC | | | | |
| --- | --- | --- | --- | --- |
| **Category** | **Sub-category** | **p-value** | **FDR** | **Enriched in** |
| Biological Process | System development (GO:0048731) | 9.95E-06 | 2.17E-02 | hDFs +MMC |
|  | Multicellular organism development (GO:0007275) | 3.66E-05 | 4.00E-02 | hDFs +MMC |
| Cellular Component | Extracellular region (GO:0005576) | 5.73E-05 | 2.91E-02 | hDFs +MMC |
|  | Extracellular space (GO:0005615) | 1.74E-04 | 2.95E-02 | hDFs +MMC |
|  | Extracellular matrix (GO:0031012) | 9.53E-05 | 2.42E-02 | hDFs +MMC |
| Protein Class | Calmodulin-related (PC00061) | 4.97E-04 | 4.80E-02 | hDFs +MMC |
|  | Extracellular matrix structural protein (PC00103) | 3.58E-04 | 6.92E-02 | hDFs +MMC |

| **Supplementary Table S6:** PANTHER statistical over-representation tests for protein hits in hTCs -MMC vs. hTCs +MMC | | | | |
| --- | --- | --- | --- | --- |
| **Category** | **Sub-category** | **p-value** | **FDR** | **Enriched in** |
| Biological Process | Regulation of multicellular organismal development (GO:2000026) | 1.62E-04 | 4.41E-02 | hTCs +MMC |
|  | Negative regulation of multicellular organismal process (GO:0051241) | 1.31E-04 | 4.07E-02 | hTCs +MMC |
|  | Haemostasis (GO:0007599) | 3.63E-06 | 7.92E-03 | hTCs +MMC |
|  | Regulation of body fluid levels (GO:0050878) | 8.88E-06 | 4.84E-03 | hTCs +MMC |
|  | Coagulation (GO:0050817) | 4.20E-06 | 3.05E-03 | hTCs +MMC |
|  | Blood coagulation (GO:0007596) | 3.63E-06 | 3.96E-03 | hTCs +MMC |
|  | Zymogen activation (GO:0031638) | 1.84E-04 | 4.46E-02 | hTCs +MMC |
|  | Response to wounding (GO:0009611) | 2.45E-05 | 8.91E-03 | hTCs +MMC |
|  | Wound healing (GO:0042060) | 2.09E-05 | 9.11E-03 | hTCs +MMC |
| Cellular Component | Extracellular region (GO:0005576) | 3.10E-15 | 1.57E-12 | hTCs +MMC |
|  | Extracellular space (GO:0005615) | 5.89E-11 | 1.50E-08 | hTCs +MMC |
|  | Extracellular matrix (GO:0031012) | 1.27E-06 | 1.61E-04 | hTCs +MMC |
|  | Collagen-containing extracellular matrix (GO:0062023) | 6.86E-07 | 1.16E-04 | hTCs +MMC |
| Molecular Function | Endopeptidase activity (GO:0004175) | 1.22E-05 | 6.78E-03 | hTCs +MMC |
|  | Serine-type endopeptidase activity (GO:0004252) | 3.64E-05 | 1.01E-02 | hTCs +MMC |
|  | Peptidase activity (GO:0008233) | 5.30E-05 | 4.91E-03 | hTCs +MMC |
|  | Serine-type peptidase activity (GO:0008236) | 4.09E-05 | 5.68E-03 | hTCs +MMC |
|  | Hydrolase activity, acting on acid phosphorus-nitrogen bonds (GO:0016825) | 4.09E-05 | 4.55E-03 | hTCs +MMC |
|  | Serine hydrolase activity (GO:0017171) | 4.09E-05 | 7.58E-03 | hTCs +MMC |
| Protein Class | Protease inhibitor (PC00191) | 3.36E-08 | 6.48E-06 | hTCs +MMC |
|  | Protein-binding activity modulator (PC00095) | 4.59E-04 | 4.43E-02 | hTCs +MMC |

| **Supplementary Table S7:** PANTHER statistical over-representation tests for protein hits in hDFs -MMC vs. hDFs +MMC | | | | |
| --- | --- | --- | --- | --- |
| **Category** | **Sub-category** | **p-value** | **FDR** | **Enriched in** |
| Protein Class | Extracellular matrix protein (PC00102) | 9.42E-05 | 1.82E-02 | hDFs -MMC |
|  | Extracellular matrix structural protein (PC00103) | 1.89E-04 | 1.82E-02 | hDFs -MMC |
|  |  |  |  |  |
| Cellular Component | Extracellular region (GO:0005576) | 1.16E-05 | 2.94E-03 | hDFs +MMC |
|  | Extracellular space (GO:0005615) | 6.34E-04 | 4.03E-02 | hDFs +MMC |
|  | Extracellular matrix (GO:0031012) | 1.26E-04 | 1.28E-02 | hDFs +MMC |
|  | Collagen-containing extracellular matrix (GO:0062023) | 7.76E-05 | 9.86E-03 | hDFs +MMC |
|  | Spliceosomal complex (GO:0005681) | 1.08E-03 | 4.98E-02 | hDFs +MMC |
|  | Nucleolus (GO:0005730) | 8.02E-04 | 4.53E-02 | hDFs +MMC |
|  | Preribosome (GO:0030684) | 1.07E-03 | 5.44E-02 | hDFs +MMC |
|  | Ribonucleoprotein granule (GO:0035770) | 1.67E-04 | 1.21E-02 | hDFs +MMC |
|  | Cytoplasmic ribonucleoprotein granule (GO:0036464) | 1.67E-04 | 1.42E-02 | hDFs +MMC |
|  | Supramolecular complex (GO:0099080) | 3.80E-05 | 6.44E-03 | hDFs +MMC |
|  | Ribonucleoprotein complex (GO:1990904) | 2.79E-07 | 1.42E-04 | hDFs +MMC |
| Molecular function | Endopeptidase activity (GO:0004175) | 7.67E-05 | 4.26E-02 | hDFs +MMC |
| Protein Class | RNA metabolism protein (PC00031) | 1.62E-05 | 3.13E-03 | hDFs +MMC |
|  | Protease inhibitor (PC00191) | 1.62E-05 | 3.13E-03 | hDFs +MMC |

| **Supplementary Table S8:** Matrisome protein hits in hTCs -MMC vs. hDFs -MMC | | | |
| --- | --- | --- | --- |
| **Matrisome division** | **Matrisome category** | **Gene Symbol** | **Enriched in** |
| Core matrisome | ECM Glycoproteins | FGL2 | hTCs -MMC |
| Core matrisome | ECM Glycoproteins | EDIL3 | hTCs -MMC |
| Matrisome-associated | Secreted Factors | TGFB2 | hTCs -MMC |
|  |  |  |  |
| Core matrisome | Collagens | COL5A1 | hDFs -MMC |
| Core matrisome | Collagens | COL16A1 | hDFs -MMC |
| Core matrisome | ECM Glycoproteins | EMILIN2 | hDFs -MMC |
| Core matrisome | ECM Glycoproteins | FBLN1 | hDFs -MMC |
| Core matrisome | ECM Glycoproteins | FBLN2 | hDFs -MMC |
| Core matrisome | ECM Glycoproteins | FBN1 | hDFs -MMC |
| Core matrisome | ECM Glycoproteins | FN1 | hDFs -MMC |
| Core matrisome | ECM Glycoproteins | MXRA5 | hDFs -MMC |
| Core matrisome | ECM Glycoproteins | PXDN | hDFs -MMC |
| Core matrisome | ECM Glycoproteins | SLIT3 | hDFs -MMC |
| Core matrisome | ECM Glycoproteins | THBS1 | hDFs -MMC |
| Core matrisome | ECM Glycoproteins | TNC | hDFs -MMC |
| Core matrisome | Proteoglycans | DCN | hDFs -MMC |
| Core matrisome | Proteoglycans | PRELP | hDFs -MMC |
| Core matrisome | Proteoglycans | VCAN | hDFs -MMC |
| Matrisome-associated | ECM Affiliated Proteins | PLXNC1 | hDFs -MMC |
| Matrisome-associated | ECM Affiliated Proteins | PLXND1 | hDFs -MMC |
| Matrisome-associated | ECM Regulators | CTSK | hDFs -MMC |
| Matrisome-associated | ECM Regulators | SERPINB2 | hDFs -MMC |
| Matrisome-associated | ECM Regulators | MMP1 | hDFs -MMC |
| Matrisome-associated | ECM Regulators | SERPINE2 | hDFs -MMC |
| Matrisome-associated | Secreted Factors | S100A4 | hDFs -MMC |

| **Supplementary Table S9:** Matrisome protein hits in hTCs +MMC vs. hDFs +MMC | | | |
| --- | --- | --- | --- |
| **Matrisome division** | **Matrisome category** | **Gene Symbol** | **Enriched in** |
| Core matrisome | Collagens | COL14A1 | hTCs +MMC |
| Core matrisome | Collagens | COL2A1 | hTCs +MMC |
| Core matrisome | ECM Glycoproteins | EDIL3 | hTCs +MMC |
| Matrisome-associated | ECM Regulators | CTSL | hTCs +MMC |
| Matrisome-associated | ECM Regulators | PLOD2 | hTCs +MMC |
| Matrisome-associated | Secreted Factors | TGFB2 | hTCs +MMC |
|  |  |  |  |
| Core matrisome | Collagens | COL16A1 | hDFs +MMC |
| Core matrisome | ECM Glycoproteins | ECM1 | hDFs +MMC |
| Core matrisome | ECM Glycoproteins | EMILIN2 | hDFs +MMC |
| Core matrisome | ECM Glycoproteins | FBLN2 | hDFs +MMC |
| Core matrisome | ECM Glycoproteins | FBLN7 | hDFs +MMC |
| Core matrisome | ECM Glycoproteins | LTBP2 | hDFs +MMC |
| Core matrisome | ECM Glycoproteins | SLIT3 | hDFs +MMC |
| Core matrisome | ECM Glycoproteins | THBS1 | hDFs +MMC |
| Core matrisome | ECM Glycoproteins | THBS2 | hDFs +MMC |
| Matrisome-associated | ECM Affiliated Proteins | PLXNC1 | hDFs +MMC |
| Matrisome-associated | ECM Regulators | CTSK | hDFs +MMC |
| Matrisome-associated | ECM Regulators | LOX | hDFs +MMC |
| Matrisome-associated | ECM Regulators | MMP1 | hDFs +MMC |
| Matrisome-associated | ECM Regulators | SERPINB2 | hDFs +MMC |
| Matrisome-associated | ECM Regulators | SERPINE2 | hDFs +MMC |
| Matrisome-associated | Secreted Factors | S100A4 | hDFs +MMC |
| Matrisome-associated | Secreted Factors | S100A6 | hDFs +MMC |
| Matrisome-associated | Secreted Factors | S100A10 | hDFs +MMC |

| **Supplementary Table S10:** Matrisome protein hits in hTCs -MMC vs. hTCs +MMC | | | |
| --- | --- | --- | --- |
| **Matrisome division** | **Matrisome category** | **Gene Symbol** | **Enriched in** |
| Core matrisome | ECM Glycoproteins | EDIL3 | hTCs -MMC |
| Core matrisome | ECM Glycoproteins | FBLN2 | hTCs -MMC |
| Core matrisome | Proteoglycans | HSPG2 | hTCs -MMC |
|  |  |  |  |
| Core matrisome | ECM Glycoproteins | CRISPLD2 | hTCs +MMC |
| Core matrisome | ECM Glycoproteins | ECM1 | hTCs +MMC |
| Core matrisome | ECM Glycoproteins | IGFBP3 | hTCs +MMC |
| Core matrisome | ECM Glycoproteins | SLIT2 | hTCs +MMC |
| Core matrisome | ECM Glycoproteins | SRPX | hTCs +MMC |
| Core matrisome | ECM Glycoproteins | THBS1 | hTCs +MMC |
| Core matrisome | ECM Glycoproteins | THBS2 | hTCs +MMC |
| Matrisome-associated | ECM Affiliated Proteins | C1QTNF3 | hTCs +MMC |
| Matrisome-associated | ECM Affiliated Proteins | C1QTNF5 | hTCs +MMC |
| Matrisome-associated | ECM Affiliated Proteins | SEMA3C | hTCs +MMC |
| Matrisome-associated | ECM Regulators | ADAMTS1 | hTCs +MMC |
| Matrisome-associated | ECM Regulators | CTSK | hTCs +MMC |
| Matrisome-associated | ECM Regulators | F13A1 | hTCs +MMC |
| Matrisome-associated | ECM Regulators | HTRA1 | hTCs +MMC |
| Matrisome-associated | ECM Regulators | ITIH2 | hTCs +MMC |
| Matrisome-associated | ECM Regulators | ITIH3 | hTCs +MMC |
| Matrisome-associated | ECM Regulators | SERPIND1 | hTCs +MMC |
| Matrisome-associated | ECM Regulators | SERPINE2 | hTCs +MMC |
| Matrisome-associated | Secreted Factors | ANGPTL2 | hTCs +MMC |
| Matrisome-associated | Secreted Factors | GDF15 | hTCs +MMC |
| Matrisome-associated | Secreted Factors | HGFAC | hTCs +MMC |

| **Supplementary Table S11:** Matrisome protein hits in hDFs -MMC vs. hDFs +MMC | | | |
| --- | --- | --- | --- |
| **Matrisome division** | **Matrisome category** | **Gene Symbol** | **Enriched in** |
| Core matrisome | ECM Glycoproteins | FBLN2 | hDFs -MMC |
| Core matrisome | ECM Glycoproteins | FBN1 | hDFs -MMC |
| Core matrisome | ECM Glycoproteins | LTBP1 | hDFs -MMC |
| Core matrisome | ECM Glycoproteins | MXRA5 | hDFs -MMC |
| Core matrisome | Proteoglycans | BGN | hDFs -MMC |
| Core matrisome | Proteoglycans | DCN | hDFs -MMC |
| Core matrisome | Proteoglycans | HSPG2 | hDFs -MMC |
| Matrisome-associated | ECM Regulators | CD109 | hDFs -MMC |
| Matrisome-associated | ECM Regulators | SERPINF1 | hDFs -MMC |
| Matrisome-associated | ECM Regulators | SERPING1 | hDFs -MMC |
| Core matrisome | ECM Glycoproteins | COMP | hDFs +MMC |
| Core matrisome | ECM Glycoproteins | CRISPLD2 | hDFs +MMC |
| Core matrisome | ECM Glycoproteins | CTGF | hDFs +MMC |
| Core matrisome | ECM Glycoproteins | CYR61 | hDFs +MMC |
| Core matrisome | ECM Glycoproteins | ECM1 | hDFs +MMC |
| Core matrisome | ECM Glycoproteins | IGFBP3 | hDFs +MMC |
| Core matrisome | ECM Glycoproteins | PCOLCE | hDFs +MMC |
| Core matrisome | ECM Glycoproteins | SLIT2 | hDFs +MMC |
| Core matrisome | ECM Glycoproteins | SRPX | hDFs +MMC |
| Core matrisome | ECM Glycoproteins | THBS1 | hDFs +MMC |
| Core matrisome | ECM Glycoproteins | THBS2 | hDFs +MMC |
| Core matrisome | ECM Glycoproteins | TNFAIP6 | hDFs +MMC |
| Matrisome-associated | ECM Affiliated Proteins | C1QTNF3 | hDFs +MMC |
| Matrisome-associated | ECM Affiliated Proteins | C1QTNF5 | hDFs +MMC |
| Matrisome-associated | ECM Affiliated Proteins | GREM1 | hDFs +MMC |
| Matrisome-associated | ECM Regulators | ADAMTS1 | hDFs +MMC |
| Matrisome-associated | ECM Regulators | BMP1 | hDFs +MMC |
| Matrisome-associated | ECM Regulators | F13A1 | hDFs +MMC |
| Matrisome-associated | ECM Regulators | HTRA1 | hDFs +MMC |
| Matrisome-associated | ECM Regulators | ITIH2 | hDFs +MMC |
| Matrisome-associated | ECM Regulators | ITIH3 | hDFs +MMC |
| Matrisome-associated | ECM Regulators | MMP1 | hDFs +MMC |
| Matrisome-associated | ECM Regulators | SERPIND1 | hDFs +MMC |
| Matrisome-associated | ECM Regulators | SERPINE2 | hDFs +MMC |
| Matrisome-associated | ECM Regulators | TIMP3 | hDFs +MMC |
| Matrisome-associated | Secreted Factors | ANGPTL2 | hDFs +MMC |
| Matrisome-associated | Secreted Factors | GDF15 | hDFs +MMC |
| Matrisome-associated | Secreted Factors | HGFAC | hDFs +MMC |

# 3. Supplementary materials and methods

## 3.1. Sample preparation for nLC-MS/MS quantitative proteomic analysis

Following 10 days of culture, cell layers were washed three times with ice-chilled HBSS, gently detached from the culture surfaces with a cell scraper in 1 ml of HBSS, recovered in 15 ml centrifuge tubes and centrifugated for 5 minutes at 400 xg. HBSS was discarded and pellets were flash-frozen in liquid N_2_ and stored at -80ºC until protein extraction. 4 % SDS in 50 mM Tris-HCl pH 7.6 was used as lysis buffer. A protease inhibitor cocktail was added to the lysis buffer at a 1:20 dilution immediately before its use. Cell pellets were disrupted in lysis buffer with the help of 3 mm stainless steel balls (Macherey-Nagel, Germany) and a vortex. After disruption, samples were heated at 95ºC for 5 minutes, and resulting volumes were passed 10 times through a 27 G needle with the aid of a syringe. After centrifugation at 16.000 xg for 20 minutes, supernatants were collected and stored at -80ºC until further processing. The total protein concentration was estimated by means of the Bradford’s assay using BSA as standard, and the concentration of all samples was adjusted to 1 μg/ml by dilution with lysis buffer. 25 μg of each sample was further processed for mass spectrometry analysis. After diluting samples at 1:2 with ultrapure water, DNA was digested with 250 U of Benzonase^®^ nuclease (Merck, Germany) for 45 minutes at 37ºC. Subsequently, cysteines were reduced with 10 mM dithiothreitol for 30 minutes at 57ºC, and further alkylated with 20 mM 2-chloroacetamide for another 30 minutes, in the dark. Samples were processed using the SP3 protocol [1] and on-bead digested overnight at 37ºC with sequencing-grade trypsin (Promega, USA), which was added in an enzyme to protein ratio of 1:50. Peptides were modified with TMTsixplex™ [2] Isobaric Label Reagent (Thermo Fisher, USA) according to the manufacturer’s instructions. For sample clean up, an OASIS^®^ HLB µElution Plate (Waters Corporation, USA) was used. Offline high-pH reverse phase fractionation was performed on an Agilent 1200 Infinity HPLC system (Agilent Technologies, USA), equipped with a Gemini^®^ C18 column (3 μm, 110 Å, 100 x 1.0 mm) (Phenomenex, USA), resulting in 12 fractions [3].

## 3.2. nLC-MS/MS quantitative proteomic analysis

Quantitative proteomic analysis was performed by nLC-MS/MS. In-line peptide fractionation was performed with an UltiMate™ 3000 RSLC nano system (Dionex Corporation, USA) equipped with an Acclaim™ PepMap™ 100 C18 trapping cartridge (5 µm, 100 Å, 0.3 x 5 mm) (Thermo Fisher, USA) and a nanoEase™ M/Z HSS T3 C18 analytical column (1.8 µm, 100 Å, 75 µm x 250 mm) (Waters Corporation, USA). A constant flow of 0.1 % formic acid in water (solvent A) at 30 µl/min was directed onto a trapping column for 6 minutes. Following this, peptides were eluted via the analytical column with a constant flow of 0.3 µl/min with increasing percentage of 0.1 % formic acid in acetonitrile (solvent B) from 2 % to 4 % in 4 min, from 4 % to 8 % in 2 min, then 8 % to 28 % for 66 min, and finally from 28 % to 40 % for another 10 min. The outlet of the analytical column was coupled directly to an Orbitrap™ Q Exactive™ Plus hybrid quadrupole mass spectrometer (Thermo Fisher, USA) using a Nanospray Flex™ Ion Source (Thermo Fisher, USA) in positive ion mode. The peptides were introduced into the mass spectrometer via a Pico-Tip^®^ Emitter (360 µm OD x 20 µm ID; 10 µm tip) (New Objective, USA) and an applied spray voltage of 2.3 kV. The capillary temperature was set at 320°C and a full mass scan was acquired with mass range 375-1200 m/z in the Fourier transform profile mode, with a resolution of 70,000. The filling time was set at maximum of 10 ms with a limitation of 3 x 10^6^ ions. Data-dependent acquisition was performed with the resolution of the Orbitrap™ set to 17,500, with a fill time of 50 ms and a limitation of 2 x 10^5^ ions. A normalized collision energy of 32 was applied, and a loop count of 20 was used with a minimum automatic gain control trigger of 2e^2^. A dynamic exclusion time of 30 seconds was used, the peptide match algorithm was set to ‘preferred’ and charge exclusion ‘unassigned’, charge states 1, 5 - 8 were excluded. MS^2^ data was acquired in profile mode with an isolation window of 0.7 m/z. IsobarQuant [4] and Mascot (v2.2.07) [5] were chosen for data processing. A Uniprot *Homo sapiens* proteome database (UP000005640) containing common contaminants and reversed sequences was used [6]. The search parameters were the following: Carbamidomethyl (C) and TMT10 (K) (fixed modifications), Acetyl (N-term), Oxidation (M) and TMT10 (N-term) (variable modifications). A mass error tolerance of 10 ppm was set for the full scan (MS1) and 0.02 Da for the MS/MS (MS2) spectra. Trypsin was selected as a protease with an allowance of maximum two missed cleavages. A minimum peptide length of seven amino acids and at least two unique peptides were required for a protein identification. The false discovery rate (FDR) on peptide and protein level was set to 0.01.

## 3.3. Bioinformatic analyses

For the analysis of data resulting from the quantitative proteomic analysis, the protein.txt output files of IsobarQuant [4] were processed using R programming language [7]. Only proteins identified with at least two unique peptides in at least two out of the three replicates were retained for downstream analysis. The signal_sum columns were used for the analysis. Batch-effects were removed using the respective function from the limma package [8]. Batch cleaned data were normalized using the vsn package [9] and missing values were imputed with the function impute (“knn” method) of the Msnbase package [10]. Limma was used again to test for differential protein abundance between different conditions. In any given comparison, proteins were considered to show differential abundance (“hits”), when a 2-fold change was detected with an FDR below 0.05.

The full proteomic dataset was functionally classified using the PANTHER Gene List Analysis Tool [11]. *Homo sapiens* was selected as organism and PANTHER GO-Slim Cellular Component, PANTHER GO-Slim Molecular Function and PANTHER Protein Class were selected as annotation sets. The lists of differentially abundant proteins were analysed using the Statistical over-representation test from PANTHER Tools [11]. *Homo sapiens* was selected as organism and the selected annotation sets were PANTHER GO-Slim Biological Process, PANTHER GO-Slim Cellular Component, PANTHER GO-Slim Molecular Function, as well as PANTHER Protein Class. The list of whole-genome *Homo sapiens* genes was selected as reference. Only over-represented categories within the different annotation sets with an FDR below 0.05 were considered statistically significant. In addition, STRING (v11.0) database [12] was used to find functional protein association networks within the lists of differentially abundant proteins of every comparison. For this purpose, a multiple proteins search was performed with each list, using *Homo sapiens* as organism. All active interaction sources were selected with a minimum required interaction score of 0.4, and “query proteins only” was selected as maximum number of interactors to show. Disconnected nodes in the network were hidden, and clustering was performed with the MCL algorithm [13], using an inflation parameter value of 1.4. Networks were exported in .tsv format and further processed with Cytoscape (v3.8.0) [14]. Only networks with at least 3 interacting nodes were selected for further analysis. For the functional annotation of each individual network, the node list of each network was analysed with Reactome (v74) gene list analysis tool [15]. The pathway size cut-off value was set to 1000 entities. Only over-represented pathways with at least 3 overlapping components with input networks and an FDR below 0.05 were considered statistically significant. Results were graphically represented and exported using Reacfoam tool [15]. The networks for which no statistically significant over-represented pathways were found after Reactome (v74) analysis, were manually annotated when possible, using the Uniprot portal [6] and or the available scientific literature. For the identification of ECM proteins in the proteomic dataset, the full list of identified proteins was analysed with the Matrisome Annotator webtool [16]. In addition, for every given comparison, the lists of differentially abundant proteins and the lists of non-differentially abundant proteins were annotated using the Matrisome Annotator webtool [16]. Venn Diagrams were created with the corresponding webtool from the Bioinformatics & Evolutionary Genomics web resource of Dr. Van De Peer Lab [17].

# 4. Supplementary references

[1] C. Hughes, S. Foehr, D. Garfield, E. Furlong, L. Steinmetz, J. Krijgsveld, Ultrasensitive proteome analysis using paramagnetic bead technology, Mol Syst Biol 10(10) (2014) 757.

[2] L. Dayon, A. Hainard, V. Licker, N. Turck, K. Kuhn, D. Hochstrasser, P. Burkhard, J. Sanchez, Relative quantification of proteins in human cerebrospinal fluids by MS/MS using 6-plex isobaric tags, Anal Chem 80(8) (2008) 2921-2931.

[3] M. Reichel, Y. Liao, M. Rettel, C. Ragan, M. Evers, A. Alleaume, R. Horos, M. Hentze, T. Preiss, A. Millar, In planta determination of the mRNA-binding proteome of arabidopsis etiolated seedlings, Plant Cell 28(10) (2016) 2435-2452.

[4] H. Franken, T. Mathieson, D. Childs, G. Sweetman, T. Werner, I. Tögel, C. Doce, S. Gade, M. Bantscheff, G. Drewes, F. Reinhard, W. Huber, M. Savitski, Thermal proteome profiling for unbiased identification of direct and indirect drug targets using multiplexed quantitative mass spectrometry, Nat Protoc 10(10) (2015) 1567-1593.

[5] D. Perkins, D. Pappin, D. Creasy, J. Cottrell, Probability‐based protein identification by searching sequence databases using mass spectrometry data, Electrophoresis 20(18) (1999) 3551-3567.

[6] The UniProt Consortium, A. Bateman, M. Martin, S. Orchard, M. Magrane, R. Agivetova, S. Ahmad, E. Alpi, E. Bowler-Barnett, R. Britto, B. Bursteinas, H. Bye-A-Jee, R. Coetzee, A. Cukura, A. Silva, P. Denny, T. Dogan, T. Ebenezer, J. Fan, L. Castro, P. Garmiri, G. Georghiou, L. Gonzales, E. Hatton-Ellis, A. Hussein, A. Ignatchenko, G. Insana, R. Ishtiaq, P. Jokinen, V. Joshi, D. Jyothi, A. Lock, R. Lopez, A. Luciani, J. Luo, Y. Lussi, A. MacDougall, F. Madeira, M. Mahmoudy, M. Menchi, A. Mishra, K. Moulang, A. Nightingale, C. Oliveira, S. Pundir, G. Qi, S. Raj, D. Rice, M. Lopez, R. Saidi, J. Sampson, T. Sawford, E. Speretta, E. Turner, N. Tyagi, P. Vasudev, V. Volynkin, K. Warner, X. Watkins, R. Zaru, H. Zellner, A. Bridge, S. Poux, N. Redaschi, L. Aimo, G. Argoud-Puy, A. Auchincloss, K. Axelsen, P. Bansal, D. Baratin, M. Blatter, J. Bolleman, E. Boutet, L. Breuza, C. Casals-Casas, E. de Castro, K. Echioukh, E. Coudert, B. Cuche, M. Doche, D. Dornevil, A. Estreicher, M. Famiglietti, M. Feuermann, E. Gasteiger, S. Gehant, V. Gerritsen, A. Gos, N. Gruaz-Gumowski, U. Hinz, C. Hulo, N. Hyka-Nouspikel, F. Jungo, G. Keller, A. Kerhornou, V. Lara, P. Le Mercier, D. Lieberherr, T. Lombardot, X. Martin, P. Masson, A. Morgat, T. Neto, S. Paesano, I. Pedruzzi, S. Pilbout, L. Pourcel, M. Pozzato, M. Pruess, C. Rivoire, C. Sigrist, K. Sonesson, A. Stutz, S. Sundaram, M. Tognolli, L. Verbregue, C. Wu, C. Arighi, L. Arminski, C. Chen, Y. Chen, J. Garavelli, H. Huang, K. Laiho, P. McGarvey, D. Natale, K. Ross, C. Vinayaka, Q. Wang, Y. Wang, L. Yeh, J. Zhang, P. Ruch, D. Teodoro, UniProt: The universal protein knowledgebase in 2021, Nucleic Acids Res 49(D1) (2021) D480-D489.

[7] R Core Team, R: A language and environment for statistical computing, R Foundation for Statistical Computing, 2020. <https://www.R-project.org/>.

[8] M. Ritchie, B. Phipson, D. Wu, Y. Hu, C. Law, W. Shi, G. Smyth, limma powers differential expression analyses for RNA-sequencing and microarray studies, Nucleic Acids Res 43(7) (2015) e47.

[9] W. Huber, A. von Heydebreck, H. Sültmann, A. Poustka, M. Vingron, Variance stabilization applied to microarray data calibration and to the quantification of differential expression, Bioinformatics 18(Suppl 1) (2002) S96-S104.

[10] L. Gatto, K. Lilley, MSnbase-an R/Bioconductor package for isobaric tagged mass spectrometry data visualization, processing and quantitation, Bioinformatics 28(2) (2012) 288-289.

[11] P. Thomas, M. Campbell, A. Kejariwal, H. Mi, B. Karlak, R. Daverman, K. Diemer, A. Muruganujan, A. Narechania, PANTHER: A library of protein families and subfamilies indexed by function, Genome Res 13(9) (2003) 2129-2141.

[12] D. Szklarczyk, A.L. Gable, D. Lyon, A. Junge, S. Wyder, J. Huerta-Cepas, M. Simonovic, N.T. Doncheva, J.H. Morris, P. Bork, L.J. Jensen, C. von Mering, STRING v11: protein–protein association networks with increased coverage, supporting functional discovery in genome-wide experimental datasets, Nucleic Acids Research 47(D1) (2018) gky1131.

[13] S. van Dongen, Graph clustering via a discrete uncoupling process, Siam J Matrix Anal A 30(1) (2008) 121-141.

[14] P. Shannon, A. Markiel, O. Ozier, N. Baliga, J. Wang, D. Ramage, N. Amin, B. Schwikowski, T. Ideker, Cytoscape: A software environment for integrated models of biomolecular interaction networks, Genome Res 13(11) (2003) 2498-2504.

[15] B. Jassal, L. Matthews, G. Viteri, C. Gong, P. Lorente, A. Fabregat, K. Sidiropoulos, J. Cook, M. Gillespie, R. Haw, F. Loney, B. May, M. Milacic, K. Rothfels, C. Sevilla, V. Shamovsky, S. Shorser, T. Varusai, J. Weiser, G. Wu, L. Stein, H. Hermjakob, P. D’Eustachio, The reactome pathway knowledgebase, Nucleic Acids Res 48(D1) (2019) D498-D503.

[16] A. Naba, K. Clauser, S. Hoersch, H. Liu, S. Carr, R. Hynes, The matrisome: In silico definition and in vivo characterization by proteomics of normal and tumor extracellular matrices, Mol Cell Proteomics 11(4) (2012) M111.014647.

[17] Y. Van de Peer, Bioinformatics and evolutionary genomics, calculate and draw custom Venn diagrams, 2021. <http://bioinformatics.psb.ugent.be/webtools/Venn/>.
